# CRISPR-Cas9 Knockout Screens Identify DNA Damage Response Pathways and *BTK* as Essential for Cisplatin Response in Diffuse Large B-Cell Lymphoma

**DOI:** 10.3390/cancers16132437

**Published:** 2024-07-02

**Authors:** Issa Ismail Issa, Hanne Due, Rasmus Froberg Brøndum, Vidthdyan Veeravakaran, Hulda Haraldsdóttir, Cathrine Sylvester, Asta Brogaard, Soniya Dhanjal, Bernhard Schmierer, Karen Dybkær

**Affiliations:** 1 Department of Hematology, Clinical Cancer Research Center, Aalborg University Hospital, 9000 Aalborg, Denmark; 2 Department of Clinical Medicine, Aalborg University, 9000 Aalborg, Denmark; 3 Center for Clinical Data Science (CLINDA), Department of Clinical Medicine, Aalborg University, and Research, Education and Innovation, Aalborg University Hospital, 9260 Gistrup, Denmark; 4 CRISPR Functional Genomics, SciLifeLab and Karolinska Institutet, Department of Medical Biochemistry and Biophysics, 17165 Solna, Sweden

**Keywords:** diffuse large B-cell lymphoma, cisplatin, CRISPR, relapse, DNA damage response, ibrutinib

## Abstract

**Simple Summary:**

Diffuse large B-cell lymphoma is the most common type of lymphoma. Despite initial treatment, up to 40% of patients do not achieve a cure and need second-line therapy. For those experiencing late relapse or lacking access to CAR-T cell therapy, platinum-based chemotherapy followed by stem cell transplantation remains the standard of care. In this study, we used genomewide CRISPR/Cas9 screens and single-gene knockout experiments to identify the genes associated with responses to platinum-based drugs. We provide a comprehensive list of genes involved in the response to cisplatin in DLBCL. Our functional experiments highlight the critical roles of the DNA damage response genes *XPA* and *ERCC6*, in addition to *BTK*, in the response to platinum-based drugs. Additionally, we show that inhibition of BTK at lower concentrations sensitizes DLBCL cells to platinum-based drugs.

**Abstract:**

The recurrence of diffuse large B-cell lymphoma (DLBCL) has been observed in 40% of cases. The standard of care for refractory/relapsed DLBCL (RR-DLBCL) is platinum-based treatment prior to autologous stem cell transplantation; however, the prognosis for RR-DLBCL patients remains poor. Thus, to identify genes affecting the cisplatin response in DLBCL, cisplatin-based whole-genome CRISPR-Cas9 knockout screens were performed in this study. We discovered DNA damage response (DDR) pathways as enriched among identified sensitizing CRISPR-mediated gene knockouts. In line, the knockout of the nucleotide excision repair genes *XPA* and *ERCC6* sensitized DLBCL cells to platinum drugs irrespective of proliferation rate, thus documenting DDR as essential for cisplatin sensitivity in DLBCL. Functional analysis revealed that the loss of *XPA* and *ERCC6* increased DNA damage levels and altered cell cycle distribution. Interestingly, we also identified *BTK,* which is involved in B-cell receptor signaling, to affect cisplatin response. The knockout of *BTK* increased cisplatin sensitivity in DLBCL cells, and combinatory drug screens revealed a synergistic effect of the BTK inhibitor, ibrutinib, with platinum drugs at low concentrations. Applying local and external DLBCL cohorts, we addressed the clinical relevance of the genes identified in the CRISPR screens. *BTK* was among the most frequently mutated genes with a frequency of 3–5%, and *XPA* and *ERCC6* were also mutated, albeit at lower frequencies. Furthermore, 27–54% of diagnostic DLBCL samples had mutations in pathways that can sensitize cells to cisplatin. In conclusion, this study shows that *XPA* and *ERCC6*, in addition to *BTK*, are essential for the response to platinum-based drugs in DLBCL.

## 1. Introduction

Diffuse large B-cell lymphoma (DLBCL) is a heterogeneous lymphoid cancer constituting 30–40% of non-Hodgkin lymphoma cases. The standard first-line treatment is the immunochemotherapy regimen consisting of rituximab, cyclophosphamide, doxorubicin, vincristine, and prednisone (R-CHOP); however, up to 40% of patients become refractory to initial therapy or relapse after treatment (RR-DLBCL) [1,2]. The standard of care for RR-DLBCL patients has been platinum-based second-line regimens, most commonly R-DHAP (rituximab with dexamethasone, high-dose cytarabine, and cisplatin), R-ICE (rituximab with ifosfamide, carboplatin, and etoposide), or R-GDP (rituximab with gemcitabine, dexamethasone, and cisplatin), followed by high-dose therapy and autologous stem cell transplantation (ASCT), has been used for transplant-eligible patients until recently. Based on positive results from clinical trials, Anti-CD19 CAR T-cell therapy has now been approved by the European Medicines Agency (EMA) and the Food and Drug Administration (FDA) for the treatment of DLBCL patients that are either refractory to first-line therapy or relapse within 12 months [3,4,5]. However, for patients relapsing later, platinum-based therapies are still the standard of care. In addition, the complex manufacturing process and expensive therapy still present as implementation barriers in many parts of the world [6]. Thus, platinum-based treatments are still widely used for RR-DLBCL patients. 

Phase 3 clinical trials have shown that there is no overall difference in the survival rates between these three platinum-based salvage regimens [7,8]. Indeed, for RR-DLBCL patients treated with platinum-based therapies, the prognosis is poor, with most being refractory or relapsing once again after platinum treatment and only 20–25% of refractory/early relapse patients being alive after 2 years after the commencement of salvage therapy [2,9,10]. 

Platinum-based drugs, such as cisplatin and carboplatin, bind to DNA purine bases guanine and adenine and form intra- and interstrand crosslinks. These platinum–DNA adducts block DNA replication and transcription and cause DNA double-strand breaks (DSBs), thus leading to excessive DNA damage and ultimately apoptosis [11]. The cytotoxic property of platinum-based drugs is exploited when treating a wide range of cancers such as ovary, breast, and lung cancer, in addition to lymphoma [11]. Effective responses to platinum-based drugs are associated with a deficiency in the DNA damage response (DDR) [12,13], which is a network of cellular pathways that sense, signal, and repair the DNA damage responsible for maintaining genomic stability [14,15]. In normal cells, surveillance proteins monitor DNA integrity and activate DNA repair pathways and cell cycle checkpoints when DNA damage is recognized, thereby shielding the cell from potential harm. However, genetic alterations in cancerous cells that interfere with the regulation or function of DDR pathways cause genome instability and DDR deficiency, thus rendering the cancer cells susceptible to DNA damage [14]. Thus, cancer cells are vulnerable and dependent on the remaining functional DDR pathways for survival [16]. 

The main DDR pathways involved in repairing platinum-induced DNA damage are nucleotide excision repair (NER) specialized in the repair of bulky DNA adducts, mismatch repair (MMR), and homologous recombination (HR), which mediates the repair of DSBs in an error-free manner [12,17]. 

Following platinum drug treatment, most bulky lesions are repaired through the global genomic–NER subpathway, in which NER surveillance proteins detect the bulky lesions and recruit transcription factor II H (TFIIH), along with XPA and RPA, to repair the damaged site. If RNA polymerase II gets stalled, it can activate the transcription-coupled NER subpathway, where it recruits ERCC6 (CSB), which coordinates repair of the DNA [18,19]. 

Despite the potent antitumor effects of platinum-based drugs, the majority of RR-DLBCL patients remain uncured, even when eligible for salvage therapy in combination with ASCT [9]. For these patients, improvements on the current salvage therapies and upfront identification of patients unlikely to achieve a durable response remain a challenge. In this regard, uncovering targetable genes and molecular pathways affecting the response to platinum treatment in DLBCL cells could provide useful insights into patient stratification and improved treatment outcomes. 

Given the poor prognosis of RR-DLBCL patients and the need for the identification of genes that sensitize DLBCL cells to platinum-based treatment, we performed whole-genome CRISPR-Cas9 knockout (KO) screens in DLBCL cells to identify the genes affecting cisplatin response in DLBCL. 

## 2. Materials and Methods

### 2.1. Cell Culturing

DLBCL cell lines—HBL1, OCILY7, RIVA, and SUDHL5—were grown in RPMI medium 1640 supplemented with fetal bovine serum and 1% penicillin–streptomycin at 37 °C in a humidified atmosphere with 5% CO_2_. Characteristics and extended information regarding cell lines are described in Table S1. All cell lines were DNA barcoded to verify cell line identity, as previously described [20], and tested for mycoplasma (Sartorius, Göttingen, Germany, Cat#SKU20-700-20) throughout the study. 

### 2.2. CRISPR Knockout Library Screening

**Figure 1:**
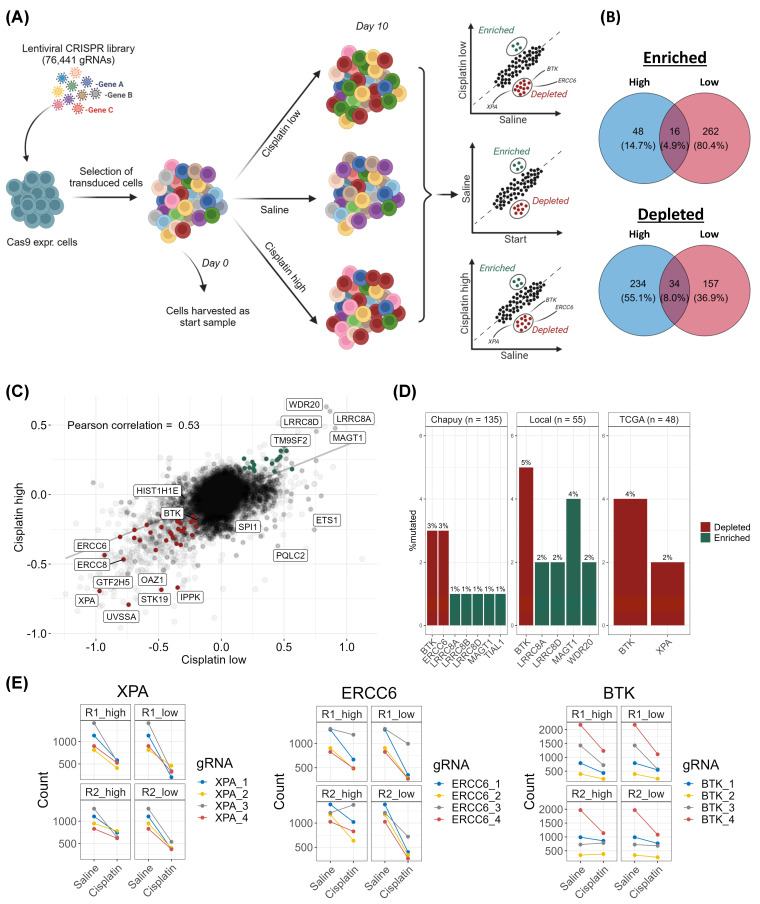
Genomewide CRISPR screens using cisplatin in DLBCL cells. (**A**) Experimental setup for the CRISPR screens using two doses of cisplatin for 10 days (created with biorender.com). (**B**) From the screens, a total of 326 genes conferring resistance to cisplatin upon knockout and 425 genes conferring sensitivity to cisplatin upon knockout were identified between the two doses. (**C**) Comparison of gene scores (β-scores) calculated using MAGeCK-MLE between the two doses of cisplatin. Pearson correlation between the two doses was assessed considering all genes (faint dots, and in center). Genes in the enriched category (green if observed in both doses or else black) and depleted category (red if observed in both doses or else black) are illustrated. (**D**) Mutational frequencies (nonsynonymous variants) of the cisplatin-sensitizing gene knockouts identified from the CRISPR screen were examined for mutation frequencies in three clinical DLBCL cohorts consisting of pre-treatment diagnostic samples. Green genes are from the enriched category, red genes are from the depleted category. (**E**) gRNA counts from the CRISPR screen of genes chosen for validation experiments. Lines illustrate gRNA-wise comparisons between the saline controls and cisplatin treated knockout populations after 10 days, in which negative slopes indicate a depletion in the cisplatin treated population.

Whole-genome CRISPR-Cas9 KO screens were performed in OCILY7 cells using the Brunello library, including an average of four gRNAs per gene (Figure 1A) [21]. In brief, stable OCILY7^spCas9^ cells were produced using lentiviral transduction with Lenti-Cas9-T2A-Blast-BFP plasmid (Addgene #196714, Watertown, MA, USA) on parental cells. Oligos generated from the genomewide Brunello sgRNA library were cloned by Gibson assembly into pLenti-Puro-AU-flip-3xBsmBI (Addgene #196709) and packaged into lentivirus using HEK-293T cells [22]. OCILY7^spCas9^ cells were transduced with the library in two replicates at a multiplicity of infection (MOI) of 0.3 and a coverage of 1000 cells per guide and selected using puromycin (2 µg/mL). For each replicate, an initial baseline sample was harvested on day 0. To ensure library coverage, 1 × 10^8^ cells of each replicate were treated with cisplatin (1.5 and 6 µg/mL cisplatin) or saline as control for 10 days. During culturing, all cells were retained when cell numbers were less than 1 × 10^8^ cells. Genomic DNA was isolated and sequenced as outlined in Schmierer et al. 2017 [22]. 

### 2.3. Clinical Cohorts

Whole-exome sequencing (WES) on diagnostic DLBCL samples from a local cohort [23] (n = 55), a published external cohort [24] (n = 135), and The Cancer Genome Atlas dataset [25] (n = 48), were used to examine mutation frequencies of CRISPR candidate genes and to explore DDR gene mutation frequencies.

### 2.4. Drug Response Assays

The following drugs were used: cisplatin and carboplatin (Aalborg University Hospital Pharmacy) in isotonic saline solutions, as well as ibrutinib (Selleckchem, Houston, TX, USA, Cat#S2680). Dose–response screens were performed as previously described [13] using the seeding concentrations listed in Table S1. Briefly, cell viability assays were performed using MTS reagent (Promega, Madison, WI, USA, Cat#G3581) by seeding cells in 96-well plates one day before adding drugs. After 48h of drug exposure, readout was performed at 490 nm absorbance (FLUOstar Omega, BMG LABTECH, Ortenberg, Germany). Viability was reported relative to saline-treated controls. Proliferation rates were determined in a similar manner without drugs using 0 h and 48 h plates. All MTS assays were run in six replicates at least three times. 

### 2.5. Generation of Single Gene Knockouts

Guide RNAs (gRNAs) were designed using CRISPOR [26] and purchased as chemically modified gRNAs (Synthego, Redwood City, CA, USA), along with the most efficient gRNAs from the CRISPR screens (Table S2). For nucleofection, ribonucleoprotein (RNP) duplexes were made using 3.2 µg gRNA mixed with 6 µg spCas9 (Integrated DNA Technologies, Clareville, IA, USA, Cat#1081061 and Cat#10008161) at 25 °C for 15 min. Setup for transfection of each cell line is detailed in Table S1. Harvested cells were washed once in either PBS or OPTI-MEM (Gibco, Grand Island, NY, USA, Cat#11058021) and resuspended in 20 µL Buffer SG (Lonza, Walkersville, MD, USA, PBC3-00675) or OPTI-MEM. The cells were mixed with RNP complexes and placed in nucleofector strips in a 4D-Nucleofector X Unit (Lonza). Electroporated cells were mixed in 100 µL growth media and cultured 10–14 days prior to harvest and subsequent functional studies. 

To validate gene KO, genomic DNA was purified, and the KO site was PCR-amplified using flanking PCR primers (Table S2). Samples were Sanger sequenced (Eurofins Genomics, 51105 Köln, Germany), and editing efficiencies were assessed using Synthego’s ICE tool [27]. Moreover, western blot was performed as outlined in Supplementary methods using products specified in Table S3.

### 2.6. DNA Damage and Cell Cycle Analysis 

Ethanol-fixed samples were analyzed using flow cytometry (SONY, Sony Corp., Tokyo, Japan, SH800 Cell Sorter) for DNA damage and cell cycle analysis, as outlined in Supplementary Materials. DNA damage was detected using γH2AX staining as previously described [13], and cell cycle distributions were detected using propidium iodide staining. 

### 2.7. Biostatistical Analysis

All statistical analyses of the drug response assays were performed using GraphPad PRISM v.10.0. One-way or two-way ANOVA analyses were applied to compare means between samples, and *p*-values were adjusted using Bonferroni multiple comparison testing. Statistical significance was set at *p* < 0.05. FCS files were analyzed using FlowJo v.10.10. Experiments were run at least three times with six technical replicates for MTS-based assays and in technical triplicates at least twice for flow cytometry-based assays. Prior to pooling, each run from the MTS-based data was ensured not to differ significantly, and data were tested for normality before ANOVA tests. 

Sequenced genomic DNA from the CRISPR screens was analyzed using the MAGeCKFlute pipeline [28]. Here, the MAGeCK robust ranking aggregation (RRA) algorithm [29] was used for the essentiality screen, where we compared baseline to day 10 saline-treated control samples, and DepMap datasets were used for comparison and filtering (https://depmap.org/). Identification of genes and pathways affecting cisplatin response was done by applying the MAGeCK-MLE approach, including data from both baseline, saline, and cisplatin-treated samples in separate models for high and low doses, respectively. We omitted common essential genes with a log-fold change <−0.4, and we defined gene KOs above ±2 standard deviations of β-scores and unaffected in saline as either enriched or depleted. 

## 3. Results

### 3.1. CRISPR-Cas9 Screens Identify DNA Damage Response Genes as Essential for Response to Cisplatin in DLBCL

As cisplatin is used in multiple second-line DLBCL therapies, the genes conferring cellular sensitivity and resistance to cisplatin exposure were investigated using whole-genome CRISPR-Cas9 KO screens (Figure 1). The screens were performed using two concentrations (1.5 and 6 µg/mL) of cisplatin to uncover potential dose-related response mechanisms. The peak plasma concentration has previously been reported to be ~6 µg/mL immediately after a bolus injection of 100 mg/mL [30].

Assessment of the CRISPR screen quality revealed a high coverage of gRNAs, a low Gini index, and a high number of mapped reads, which document high quality (Figure S1A–G). The specific gRNA count data can be found in Table S4. The replicates, R1 and R2, showed high a Pearson correlation of 0.86 when comparing the log2-fold changes of gRNA counts between the baseline and day 10 saline samples (Figure S2A), where most gRNAs were unaffected or depleted. 

First, using the CRISPR screen data, an essential gene analysis was conducted by comparing the frequencies of gRNAs in the baseline sample (day 0) to saline-treated controls at day 10. In this regard, gRNAs targeting essential genes required for cellular survival will be depleted over time. Pathway enrichment analysis of the identified depleted gRNAs revealed MYC targets, among others, as essential for cellular survival in OCILY7 (Figure S2B,C). As the screens were restricted to OCILY7, the data from DepMap were included to validate them and to overcome the potential bias of cell line-specific essential genes. The CRISPR gene scores in our screens were compared to all cell lines in DepMap in addition to a lymphocyte subset, and correlations of 0.59–0.65 were observed (Figure S2D,E). Here, less depletion (defined as negative gene scores and FDR < 0.05, Table S5A,B) was observed in our CRISPR screens, thus resulting in fewer identified essential genes compared to the common essential genes from DepMap [31,32] (Figure S2F). In addition, for R1 and R2, 60.9–62.2% of the depleted gene KOs could be found in the two common essential gene lists (Figure S2F). Using this information and to account for cell line-specific essential genes, an analysis of the cisplatin-treated populations was conducted using MAGeCK-MLE in which DepMap common essential genes were omitted.

From the CRISPR screen, gene KOs leading to cisplatin sensitivity and resistance were identified. In total for both doses, gRNAs targeting 425 genes were depleted; when these genes are knocked out, the cells become sensitive to cisplatin and die, thus resulting in a depletion of the gRNAs from the cell population. Additionally, the enrichment of gRNAs targeting 326 genes was identified, thus defining the genes in which KO causes cisplatin resistance (Figure 1B,C and Figure S3, Tables S6A–C and S7A–C). Between the two cisplatin doses applied for selection, only 34 (8%) gene KOs were depleted in both, and only 16 (4.9%) gene KOs were enriched in both doses (Figure 1B, Table S8). Among the most enriched gRNA target genes were *LRR8B* observed in both doses and *LRRC8A* and *LRRC8D* observed at the highest dose (Figure 1C). These genes encode subunits of the volume-regulated anion channel (VRAC), which is responsible for up to 50% of platinum uptake, and the KOs of these genes are commonly identified to confer resistance in platinum drug-based CRISPR screens [12,33], thereby serving as positive controls for successful screening and drug treatment. Pathway analysis of the cisplatin-sensitizing gene KOs revealed an overrepresentation of DDR pathways, especially at the low-cisplatin concentrations (Figure S4A–D). Notably, the pathways identified upon low cisplatin exposure belong to the NER, Fanconi anemia (FA), and HR pathways, whereas NER was the only DDR pathway identified upon high-dose cisplatin exposure (Figure S4A–D), suggesting dose-dependent cisplatin response mechanisms. In addition, the gRNAs targeting *EXO1*, which are involved in HR and MMR, were depleted upon high-dose cisplatin exposure.

In the cisplatin CRISPR screen, the gRNAs targeting the NER genes *XPA*, *ERCC6*, and *UVSSA* were among the most depleted across both doses of cisplatin, thus demonstrating that these genes are essential for the response to cisplatin (Figure 1B,C and Figure S3, Tables S6C and S7C). 

### 3.2. Examination of Mutation Frequencies in Clinical Cohorts and Gene Selection for Validation

For clinical relevance, the mutation frequencies of cisplatin candidate genes identified from the CRISPR screens were examined in three clinical DLBCL cohorts, so only genetic variants observed in primary DLBCL tumors have been included for functional validation. Among the most frequently mutated cisplatin candidate genes was *BTK* (3–5%) (Figure 1D), which can be targeted using the clinically relevant inhibitor, ibrutinib. The DDR genes, *XPA* and *ERCC6*, were also mutated, albeit at lower frequencies (Figure 1D). *BTK* was selected for experimental validation based on its clinical relevance along with *XPA* and *ERCC6* due to their strong cisplatin-sensitizing effect when knocked out in the CRISPR screens (Figure 1E).

To choose cell lines for validation and functional assessment, we considered the mutation status, gene expression levels, and copy number of candidate genes (Figure S4E), in addition to their DLBCL subgroup classification and transfection efficiencies. This data were gathered from the DepMap database, which does not include the HBL1 cell line. The genes chosen for validation were assessed for mutations within cell lines, pseudogenes, SNPs, technical feasibility in validating knockouts (e.g., primers, antibodies, and gRNA), and their relation to platinum drugs in the literature. Drug concentrations spanning the IC50 and preferably covering the entire dose–response curves across various cell lines were chosen for validation. 

### 3.3. XPA^KO^ and ERCC6^KO^ Confers Sensitivity to Platinum Drugs Irrespective of Proliferation Rate

**Figure 2:**
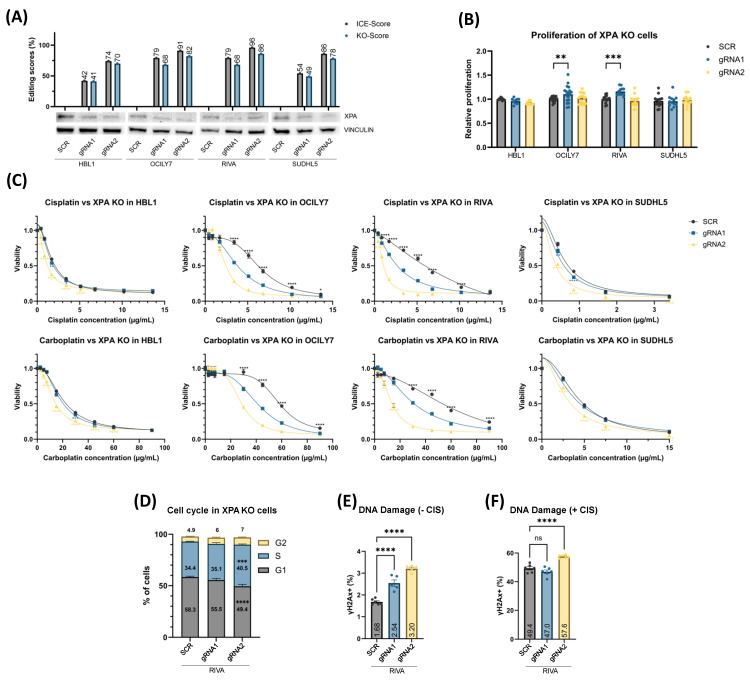
*XPA* gene knockout. (**A**) Polyclonal populations of *XPA^KO^* cells were generated using targeting ribonucleoprotein (RNP) complexes alongside scramble control gRNAs and validated using western blot. Two gRNAs were used to generate knockout in each cell line, and the effect was compared to nontargeting scramble (SCR) controls. Gray bars show the ICE scores, i.e., the percentage of indel mutations in the population, whereas the blue bars show the knockout scores, i.e., gene edits leading to frameshifts and ≥21 bp deletion. (**B**) Proliferation rate was not affected in most *XPA^KO^* cells. RIVA *XPA^KO^* were used for functional validation. (**C**) MTS-based dose–response screens showed increased sensitivity to platinum compounds in *XPA^KO^* cells across four DLBCL cell lines representing different molecular subtypes. If both gRNA display similar significance when compared to SCR, they are marked with black symbols and placed above SCR curves, and gRNA-specific significance symbols are placed under the respective curves. (**D**) Cell cycle analysis performed with propidium iodide in RIVA. (**E**,**F**) DNA damage measured using phosphorylation of H2AX (γH2AX) as a marker in RIVA *XPA^KO^* cells without (**E**) and with (**F**) cisplatin. Values are displayed as mean ± SEM (one-way or two-way ANOVA: n = 9–18 for (**B**), n = 18–24 for (**C**), and n = 6–8 for (**D**–**F**); ns *p* >  0.05; * *p*  ≤  0.05; ** *p*  ≤  0.01; *** *p*  ≤  0.001; **** *p*  ≤  0.0001 compared with SCR controls) and are summarized based on three independent experiments for (**B**,**C**) and two independent experiments for (**D**,**F**). The uncropped bolts are shown in Supplementary Materials File S1.

To validate genes whose KOs sensitized DLBCL cells to cisplatin in the CRISPR screen, we performed single-gene KO experiments in HBL1, OCILY7, RIVA, and SUDHL5, thus representing different molecular subgroups of DLBCL. Using two *XPA-targeting* gRNAs, indel scores of 42–96% and KO scores of 41–86% were achieved, with higher editing efficiencies using gRNA2 (Figure 2A, top). The KO of *XPA* was validated by western blotting (Figure 2A, bottom). The proliferation rate was not affected in HBL1 or SUDHL5 KO cells using either gRNA, whereas gRNA1 in OCILY7 and RIVA displayed a 10–16% increase in the proliferation rates (Figure 2B). When exposed to cisplatin, *XPA^KO^* cells displayed a significant increase in sensitivity (Figure 2C), which is in agreement with the CRISPR screens and was irrespective of the proliferation rates. In addition, the *XPA^KO^* cells were treated with carboplatin, which is also used in the treatment of RR-DLBCL patients, and we observed similar sensitizing effects, thus indicating that the response is not cisplatin-specific but to platinum drugs in general. The effect was most noticeable in OCILY7 and RIVA, both of which were initially the most resistant to the platinum drugs among the cell lines used.

**Figure 3:**
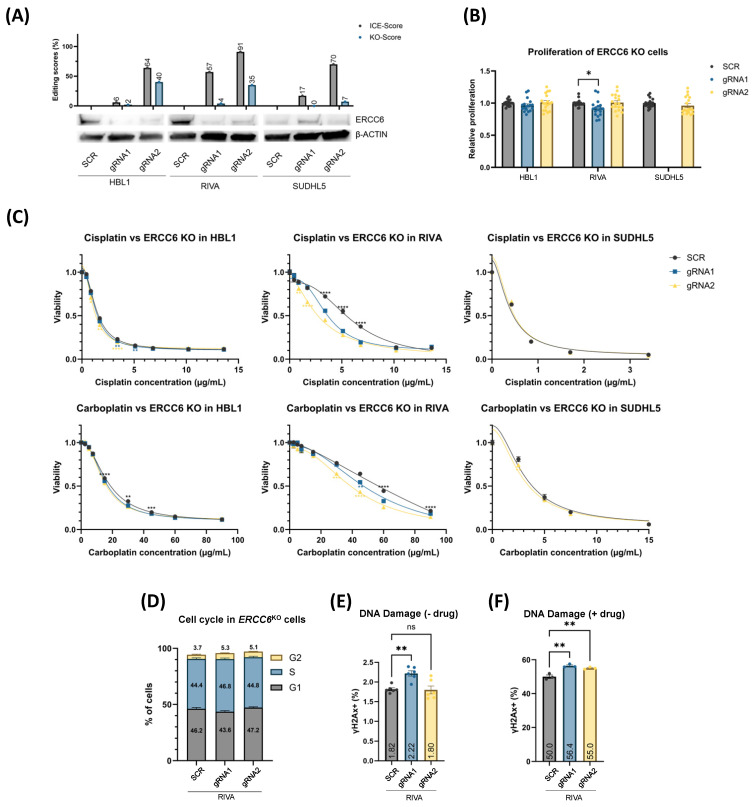
*ERCC6* gene knockout. (**A**) Polyclonal populations of *ERCC6^KO^* cells were generated using targeting ribonucleoprotein (RNP) complexes alongside scramble control gRNAs and validated using western blot. Two gRNAs were used to generate knockout in each cell line, and the effect was compared to nontargeting scramble (SCR) controls. Gray bars show the ICE scores, i.e., the percentage of indel mutations in the population, whereas the blue bars show the knockout scores, i.e., gene edits leading to frameshifts and ≥21 bp deletion. (**B**) Proliferation rate was not affected in most *ERCC6^KO^* cells. RIVA *ERCC6^KO^* were used for functional validation. (**C**) MTS-based dose–response screens showed increased sensitivity to platinum compounds in *ERCC6^KO^* cells across three DLBCL cell lines representing different molecular subtypes. If both gRNA display similar significance when compared to SCR, they are marked with black symbols and placed above SCR curves, and gRNA-specific significance symbols are placed under the respective curves. (**D**) Cell cycle analysis performed with propidium iodide in RIVA. (**E**,**F**) DNA damage measured using phosphorylation of H2AX (γH2AX) as a marker in RIVA *ERCC6^KO^* cells without (**E**) and with (**F**) cisplatin. Values are displayed as mean ± SEM (one-way or two-way ANOVA: n = 12–18 for (**B**), n = 18–24 for (**C**), and n = 6–8 for (**D**–**F**); ns *p* >  0.05; * *p*  ≤  0.05; ** *p*  ≤  0.01; *** *p*  ≤  0.001; **** *p*  ≤  0.0001 compared with SCR controls) and are summarized based on three independent experiments for (**B**,**C**) and two independent experiments for (**D**–**F**). A representative example is shown in (**D**,**F**). The uncropped bolts are shown in Supplementary Materials File S1.

Despite lower editing scores for *ERCC6^KO^* cells (indel scores 6–91% and KO scores of 2–40%), we observed similar results to *XPA^KO^* (Figure 3). In this regard, the cell lines HBL1, RIVA, and SUDHL5 were chosen to validate the sensitizing effect of *ERCC6^KO^* from the CRISPR screens. *ERCC6^KO^* was able to sensitize RIVA and, to a lesser degree, HBL1 to platinum treatment; however, no difference was observed in SUDHL5, possibly due to high initial sensitivity combined with low editing scores (Figure 3C). Thus, *XPA^KO^* and *ERCC6^KO^* had, in most cases, no impact on the DLBCL cell proliferation, but it sensitized cells to platinum treatment irrespective of the proliferation rate, with the most prominent impact on cell lines and with the least initial sensitivity to platinum drug treatment.

### 3.4. XPA^KO^ and ERCC6^KO^ Affect DNA Damage Response and Cell Cycle Distribution

Since both *XPA* and *ERCC6* are involved in DNA damage repair, we sought to investigate whether the KOs of these NER genes affect cell cycle checkpoints and impair the cells’ ability to respond to DNA damage induced through platinum drug treatment. Since the most prominent impact of *XPA* and *ERCC6* KO was observed in RIVA, these analyses were restricted to this cell line, and the phosphorylation of H2AX (γH2AX) was used as a marker of double-stranded DNA damage. In untreated cells, we did indeed observe increased S-phase arrest for the more effective gRNA2-*XPA^KO^*, in addition to increased inherent DNA damage (along with gRNA1-*XPA^KO^*) (Figure 2D,E). *ERCC6^KO^* did not affect cell cycle distributions; however, gRNA1 showed higher DNA damage levels (Figure 3D,E). Treatment with cisplatin resulted in further increases in DNA damage for both *XPA^KO^* and *ERCC6^KO^* cells when compared to control cells, possibly due to the pre-existing DNA damage and increased S-phase arrest (Figure 2F and Figure 3F).

In summary, the NER DDR genes identified in the CRISPR screens could be validated across multiple subtypes of DLBCL cells as essential for platinum-based drug response, most likely due to their inability to repair damage caused by the DNA-targeting platinum-based drugs. 

### 3.5. BTK^KO^ and Chemical Inhibition, Using Ibrutinib, Sensitize DLBCL Cells to Platinum Drugs 

To experimentally validate *BTK*, identified from the CRISPR screens to affect cisplatin response, KOs and chemical inhibition were performed. 

**Figure 4:**
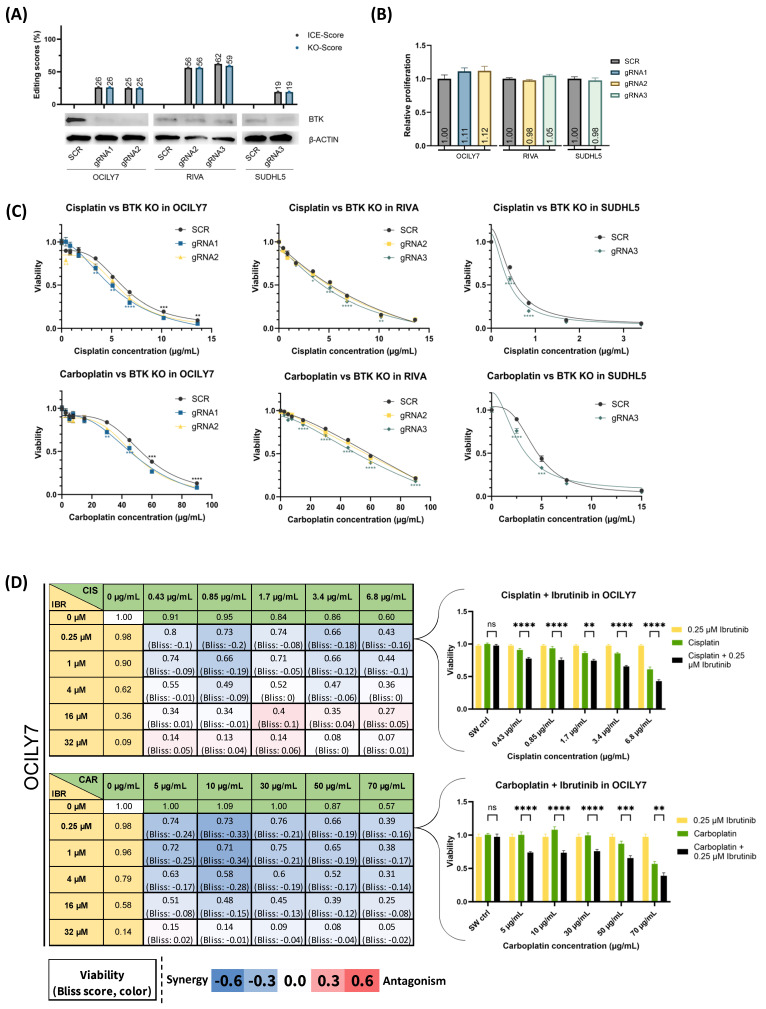
*BTK* gene knockout. (**A**) Polyclonal populations of *BTK^KO^* cells were generated using targeting ribonucleoprotein (RNP) complexes alongside scramble control gRNAs and validated using western blot. A total of 1–2 gRNAs was used to generate knockout in each cell line, and the effect was compared to nontargeting scramble (SCR) controls. Gray bars show the ICE scores, i.e., the percentage of indel mutations in the population, whereas the blue bars show the knockout scores, i.e., gene edits leading to frameshifts and ≥21 bp deletion. (**B**,**C**) MTS-based proliferation and dose–response screens across three *BTK^KO^* DLBCL cell lines. If both gRNA display similar significance when compared to SCR, they are marked with black symbols and placed above SCR curves, and gRNA-specific significance symbols are placed under the respective curves. (**D**) Drug combination screening in OCILY7 with mono and combination treatments of platinum drugs + ibrutinib leading to a total of 25 distinct combinations for cisplatin + ibrutinib (**top**) and carboplatin + ibrutinib (**bottom**). Synergistic drug interactions (negative Bliss scores, blue) and antagonistic drug interactions (positive Bliss scores, red) are displayed in each matrix figure. On the right side, bar plots display the lowest ibrutinib dose in combination with all five platinum drug doses. Values are displayed as mean ± SEM (one-way or two-way ANOVA: n = 18–24 for (**C**) and n = 12–16 for (**D**); ns *p* >  0.05; * *p*  ≤  0.05; ** *p*  ≤  0.01; *** *p*  ≤  0.001; **** *p*  ≤  0.0001 compared to SCR controls in (**C**) and to the combination treatment in (**D**)). Data are summarized based on 3–4 independent experiments for (**B**–**D**). The uncropped bolts are shown in Supplementary Materials File S1.

*BTK* was knocked out in OCILY7, RIVA, and SUDHL5, with initial KO scores between 19–59% (Figure 4A). Although not significant, a slight increase of 11–12% in the proliferation rate was observed for the OCILY7 *BTK^KO^* polyclonal cell population, which could explain the increased KO scores (Figure 4B). The *BTK^KO^* cells were treated with platinum-based drugs and displayed increased sensitivity to platinum treatment for all used cell lines (Figure 4C), albeit to a lower degree than *XPA^KO^* and *ERCC6^KO^*. Based on this drug response and to validate the association between *BTK* and platinum drugs, we decided to use the clinically relevant BTK inhibitor, ibrutinib, in combination with platinum drugs. We used doses covering the dose–response landscape to assess drug interactions. All four cell lines were treated with the respective platinum compounds or ibrutinib alone and in a 25-drug combination matrix, where drug interactions were assessed using the Bliss independence model. Here, we observed synergistic drug interactions when combining low concentrations of ibrutinib and platinum compounds (negative Bliss scores, blue) in DLBCL cell lines with inter cell line variability of synergism. These data demonstrate that chemical BTK inhibition is sufficient to drive the phenotypic impact on the platinum response (Figure 4D for OCILY7 and Figure S5A,B for all cell lines). 

### 3.6. Mutations in DNA Damage Response Genes in Diagnostic DLBCL Patients

As multiple DDR pathways were found to be involved in the cisplatin response in the CRISPR screen, we checked how many patients had mutations in the NER, MMR, or FA pathway genes, as these might be more sensitive to platinum drug treatments. In the three tested, 27–54% of the patients had mutations in the NER, MMR, or FA genes overall (Figure S6). As previously shown, *XPA* was only mutated in the TCGA cohort (2%), and *ERCC6* was mutated in the Chapuy cohort (3%) (Figure 1D and Figure S6). Although individual DDR genes were mutated at very low frequencies within the cohorts, a substantial number of patients did indeed have nonsynonymous mutations in DDR pathways known to affect response to platinum-based drugs.

## 4. Discussion

The prognosis of RR-DLBCL patients is poor, with most patients remaining refractory or developing relapse after second-line platinum-based treatment. In this study, we investigated the genes that determine the response to cisplatin in DLBCL by conducting whole-genome CRISPR-Cas9 KO screens in DLBCL cells. To the best of our knowledge, this is the first CRISPR screen using platinum drugs in lymphomas. We showed that multiple DDR pathways are essential for the response to platinum drugs, that targeting the NER pathway potentiates the effects of platinum drugs regardless of the DLBCL subtype, and that BTK can be targeted to sensitize DLBCL cells to cisplatin and carboplatin. 

DNA damage is especially of interest in B-cell lymphoma, as the B-cell in the differentiation process experiences double-strand DNA breaks, as well as intentional mutations in antibody-related genes during V(D)J recombination, class switch recombination, and somatic hypermutation [34,35]. This necessitates tolerance to DNA damage, which is strategically targeted by DNA-damaging agents. The deregulation of this process and DNA repair deficiencies are believed to promote lymphomagenesis, as it leads to genome instability and chromosomal translocations [36,37]. Targeting DDR-deficient cancer cells through synthetic lethality is used in, e.g., breast and ovarian cancers, through PARP inhibition, as the cancer cells rely more on specific DDR pathways [16]. For DLBCL patients with existing DDR deficiencies, combining DNA damaging drugs with DDR targeting inhibitors could be a feasible treatment strategy, as DDR mechanisms are frequently deregulated in lymphomas [38]. The inhibition of the NER protein, XPB, has been shown to increase sensitivity towards alkylating agents in multiple myeloma [39]. In this study, we showed that targeting multiple components of the DNA damage response pathways sensitizes DLBCL cells to platinum drugs. When the NER genes *XPA* and *ERCC6* were knocked out, the cells acquired an increased amount of baseline DNA damage, which was further increased during platinum drug exposure, thus resulting in increased sensitivity. This is in agreement with previous studies showing that *XPA* depletion increases phosphorylation of H2AX in glioblastoma and bladder cancer [40,41]. Uniquely, XPA is involved in both transcription-coupled NER and genomic NER, and strides have been taken towards the development of chemical inhibition using small molecules [42]. Effective and safe XPA inhibitors could be promising candidates for combination with platin-based treatment in DLBCL.

When performing CRISPR KO studies, DNA damage introduced by the gRNA-directed Cas9 enzyme could impact the response to DNA-damaging agents such as cisplatin, and comparison to an SCR control without Cas9-induced DSBs introduces the risk of data misinterpretation. To overcome this, transient RNP delivery was applied for generating KO cells, and in addition, functional studies were conducted at least 10 days post nucleofection giving cells time to recover. 

A previous CRISPR KO screen using cispIatin in urological cancer showed that the loss of *MSH2* (a MMR gene) increases resistance to cisplatin [43]. Like NER, MMR involves the identification, excision, resynthesis, and ligation of the synthesized strand. MSH2 is involved in the recognition of DNA damage, whereas EXO1 is involved in DNA excision. Although MMR is able to recognize platin-caused DNA lesions, it cannot repair them, whereby it engages in a futile cycle of DNA repair, thus leading to more DNA damage while also blocking NER proteins from access and resulting in increased cisplatin lethality [17]. Thus, contrary to other DDR pathways, MMR deficiency leads to resistance to platinum drugs. The only MMR gene observed in our screens was *EXO1*, which showed strong cisplatin sensitivity at both doses. However, *EXO1* is also involved in the HR DDR pathway, and downregulation has been shown to sensitize cancer cells to cisplatin specifically through the HR pathway [44]. 

Utilizing WES data from local diagnostic DLBCL patients, we observed that 27–54% of patients had nonsynonymous mutations in NER, MMR, or FA genes prior to first-line treatment. It would be highly relevant to examine mutation and gene expression data from large cohorts of RR-DLBCL tumors obtained prior to platin-based treatment to determine whether *XPA* and *ERCC6* can serve as prognostic biomarkers for the clinical outcomes of RR-DLBCL patients. In other cancers with tumor samples taken prior to cisplatin treatment, a high expression of *XPA* has been correlated with decreased OS [45,46]. Based on our findings from functional in vitro assays, it could be suspected that early treatment intervention with platinum drugs in patients with DDR mutations or low DDR-expressing tumors could impact treatment outcome positively.

Through clinical filtration of the cisplatin candidate genes identified in the CRISPR KO screens, *BTK* was observed to be mutated in up to 5% of DLBCL patients. BTK (Bruton’s tyrosine kinase) is encoded in the X chromosome and plays a vital role in B-cell receptor (BCR) signaling, where it regulates cell proliferation, differentiation, and survival. Subsequent to activation of the BCR, BTK can lead to NF-kappa-B activation, which promotes B-cell survival and proliferation [47]. 

Although DLBCL is described as one disease entity, its high heterogeneity has allowed for the identification of different molecular subtypes with prognostic differences. These are the activated B-cell-like (ABC) and germinal center B-cell-like (GCB), which make up the majority of DLBCL cases, the former of which is associated with worse prognosis [48]. ABC DLBCLs are more dependent on chronic BCR signaling to promote their growth [47]. Interestingly, we found that the KO of *BTK* sensitized both ABC and GCB DLBCL cells to platinum-based drugs, and cisplatin exposure resulted in a depletion of gRNAs targeting *BTK*, which is involved in BCR pathway signaling. To assess the clinical utility of this finding, the BTK inhibitor ibrutinib was used, which displayed synergism with the platinum-based drugs at lower concentrations. In agreement, combinatory treatment with cisplatin and ibrutinib showed synergism in ovarian cancer cells [49] and in oral squamous cell carcinoma tumorspheres, where they displayed strong tumor suppression and higher survival rates in xenograft mouse models [50]. 

Platinum drugs and ibrutinib have nonoverlapping mechanisms of action; thus, the slightly positive Bliss scores observed at higher concentrations in some cases could be caused by either one of the drugs driving the cytotoxicity within the tested timeframe, possibly combined with cell cycle aberrations. In this regard, the additive effects of drugs with nonoverlapping mechanisms of action could be sufficient to achieve increased cytotoxicity, in addition to preventing drug-specific resistance, as is shown for R-CHOP [51]. Regardless, the mechanisms behind the synergy between platinum drugs and low-dose ibrutinib treatment remains to be explored. 

Ibrutinib is already used in the treatment of chronic lymphocytic leukemia and mantle cell lymphoma. In DLBCL, the phase 3 PHOENIX trial showed that younger patients with diagnostic ABC DLBCL had an improved survival when adding ibrutinib to R-CHOP [52]. Perhaps using ibrutinib combined with platinum-based drugs for RR-DLBCL patients could be relevant for some molecular subtypes of DLBCL. 

In addition to BCR, genetic aberrations of MYC impact the prognosis in DLBCL. Patients with GCB DLBCL have higher complete remission rates when treated with R-DHAP than non-GCB, except when *MYC* gene rearrangements are present, where the outcome was worse regardless of subtype [53]. *MYC* is a proto-oncogene controlling the cell growth, survival, and cell cycle, among other cellular functions, and between 5–20% of DLBCL patients harbor MYC rearrangements that lead to unregulated overexpression and an aggressive disease course [53,54]. In our essentiality screen, GSEA analysis revealed that MYC targets are among the most depleted gene KOs, thus highlighting the oncogenic role of MYC in DLBCL. Likewise, *MYC* is among the list of common essentials and has been grouped among DLBCL essential genes in another CRISPR screen [55]. In this regard, targeting *MYC*-mediated resistance could perhaps sensitize DLBCL patient tumors to platinum drugs.

## 5. Conclusions

From these whole-genome CRISPR/Cas9 screens, we have generated lists of genes affecting the response to cisplatin, which can further be explored. Among these, we validated *XPA*, *ERCC6*, and *BTK* in this study; however, other genes in the lists are supported by the literature. Platinum drug regimens are still the backbone of RR-DLBCL treatment, and improvements in these therapies are essential for improved patient survival. The findings from this study contribute to our knowledge of the genes involved in the response to platinum-based drugs. 

## Data Availability

All analyzed data generated from the CRISPR/Cas9 screen during this study are included in this published article and its supplementary information files. The DepMap data used for comparison and filtering can be found at https://depmap.org/. The data from somatic tumors and raw in vitro data that support the findings of this study are available upon request from the corresponding author.

## Supplementary Materials

The following supporting information can be downloaded at: https://www.mdpi.com/article/10.3390/cancers16132437/s1, File S1: Original Western blot gels; Figure S1: Quality control using raw gRNA counts; Figure S2: Essentiality screen and DepMap comparison using the MAGeCK Flute library; Figure S3: Cisplatin CRISPR screen results in DLBCL cells; Figure S4: Cisplatin CRISPR screen analysis; Figure S5: BTK and platinum drug interactions; Figure S6: Oncoplot showing the frequency of DDR mutations in our local cohort; Table S1: Cell line characteristics; Table S2: sgRNAs and primers; Table S3: Western blot products; Table S4: Guide count data; Table S5: (A,B) MAGeCK-RRA results vs. DepMap data; Table S6: (A–C) MAGeCK-MLE results for low dose cisplatin; Table S7: (A–C) MAGeCK-MLE results for high dose cisplatin; Table S8: Overlapping candidate genes between both doses of cisplatin in the CRISPR screen; Supplementary materials and methods.

## Abbreviations

DLBCL	Diffuse large B-cell lymphoma
DDR	DNA damage response
DSBs	Double-strand breaks
gRNA	Guide RNA
HR	Homologous recombination
MMR	Mismatch repair
NER	Nucleotide excision repair
R-DHAP	Rituximab with dexamethasone, high-dose cytarabine, and cisplatin
R-GDP	Rituximab with gemcitabine, dexamethasone, and cisplatin
R-ICE	Rituximab with ifosfamide, carboplatin, and etoposide
RRA	Robust ranking aggregation
RR-DLBCL	Refractory/relapsed DLBCL
