# Supplementary material for: CRISPR-Cas9 Knockout Screens Identify DNA Damage Response Pathways and BTK as Essential for Cisplatin Response in Diffuse Large B-Cell Lymphoma
Source: Cancers (Basel). 2024 Jul 2;16(13):2437. doi: 10.3390/cancers16132437 (PMC11240649; doi:10.3390/cancers16132437)
Supplement: Supplementary file 1 [file cancers-16-02437-s001.zip › Supplementary figure captions.pdf]

Figure S1. Quality control using raw gRNA counts. (**A**) Median-normalized gRNA read count distribution of all samples. (**B)** Distribution of read counts. (**C**) Principal Component Analysis (PCA) of samples. (**D**) Sample clustering heatmap showing relatedness between samples. (**E**) Gini index of read count distributions, with low values indicating greater evenness of gRNA read count distribution. (**F**) Percentages of reads that can be mapped to the gRNA library (total gRNAs = 77441). (**G**) Table summarizing read counts and statistics of above figures.

Figure S2. Essentiality screen and DepMap comparison using the MAGeCK Flute library. (**A**) The two replicate (R1 and R2) screens showed a Pearson correlation of 0.86 when comparing the log2 fold changes (LFC) of gRNA counts between baseline and day 10 samples. Replicate-wise analysis of essentiality screen: (**B**) Volcano plots (top) showing distribution of each gene's score (here, log2 fold change of each gene) and -log10(FDR). Colors indicate genes with an FDR<0.05. Gene set enrichment analysis was performed using the negative LFC values and enrichment scores are plotted. (**C**) Gene Set Enrichment Analysis (GSEA) using the hallmark gene sets on the gene scores. (**D**,**E**) Gene scores in our screen vs average Gene scores for all (**D**) and lymphocyte (**E**) lineages in DepMap. Horizontal and vertical dashed lines are set to ±1.5 SD for R1/R2 and for DepMap, respectively. (**F**) Venn diagrams showing overlaps between our essential genes (defined by negative scores and FDR<0.05) and two DepMap essentiality screens.

Figure S3. Cisplatin CRISPR screen results in DLBCL cells. Gene scores (β-scores) calculated using MAGeCK-MLE were compared between cisplatin high- (left figure)/low- (right figure) concentrations and saline. Enriched (green) and depleted (orange) gRNA target genes were defined as genes above ±2 standard deviation of β-score distribution within each cisplatin screen which were not affected with saline treatment (within ±2 standard deviation of β-score distribution).

Figure S4. Cisplatin CRISPR screen analysis. **(A**,**B)** REACTOME and KEGG pathway analysis of the depleted gRNAs showed high enrichment for various DNA damage repair pathways when treated with a high (**A**) and low (**B**) dose of cisplatin. (**C**) NDEx IQuery pathway analysis of depleted gRNAs (only low dose since it contained the highest amount of DDR genes). (**D**) Utilizing a list of 276 DDR genes from *Knijnenburg et. al. 2018* we identified how many of the depleted gRNA target genes were in the DDR pathways and which doses contributed the most. (**E**) Using DepMap data, gene expression (left) and copy number (right) of genes chosen for validation was assessed in our accessible in-house cell lines. Note, HBL1 was not available in the DepMap dataset.

Figure S5. *BTK* and platinum drug interactions. (**A**,**B**) Drug combination screening in four DLBCL cell lines involved mono- and combination treatments of platinum drugs + ibrutinib. Here, five doses of each drug were used, leading to a total of 25 distinct combinations for cisplatin + ibrutinib (**A**) and carboplatin + ibrutinib (**B**). Synergistic drug interactions (negative bliss scores, blue) and antagonistic drug interactions (positive bliss scores, red) are displayed in each matrix figure. On the right side, graphs display the lowest ibrutinib dose in combination with all five platinum drug doses. Values are displayed as mean ± SEM and are representative of three independent experiments.

Figure S6. Oncoplot showing the frequency of mutations for Nucleotide excision repair (NER), Mismatch repair (MMR), and Fanconi Anemia (FA) genes in our local cohort (n=55) (**A**), the Chapuy cohort (n=135) (**B**), and the TCGA cohort (n = 48) (**C**).
