# Supplementary material for: CRISPR-Cas9 Knockout Screens Identify DNA Damage Response Pathways and BTK as Essential for Cisplatin Response in Diffuse Large B-Cell Lymphoma
Source: Cancers (Basel). 2024 Jul 2;16(13):2437. doi: 10.3390/cancers16132437 (PMC11240649; doi:10.3390/cancers16132437)
Supplement: Supplementary file 1 [file cancers-16-02437-s001.zip › Supplementary figures 30062024.pdf]

Figure S1

(A)

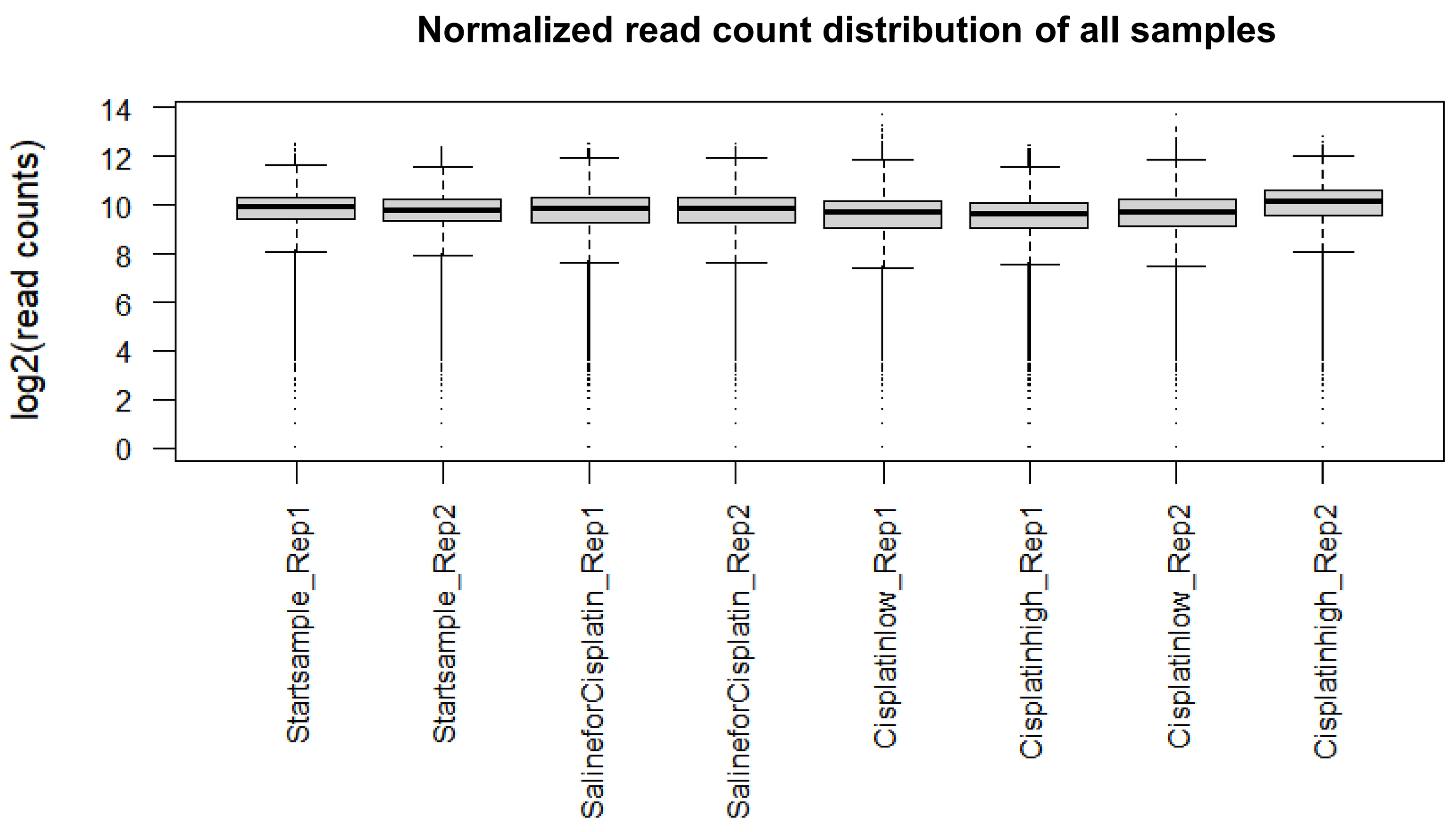

(D)

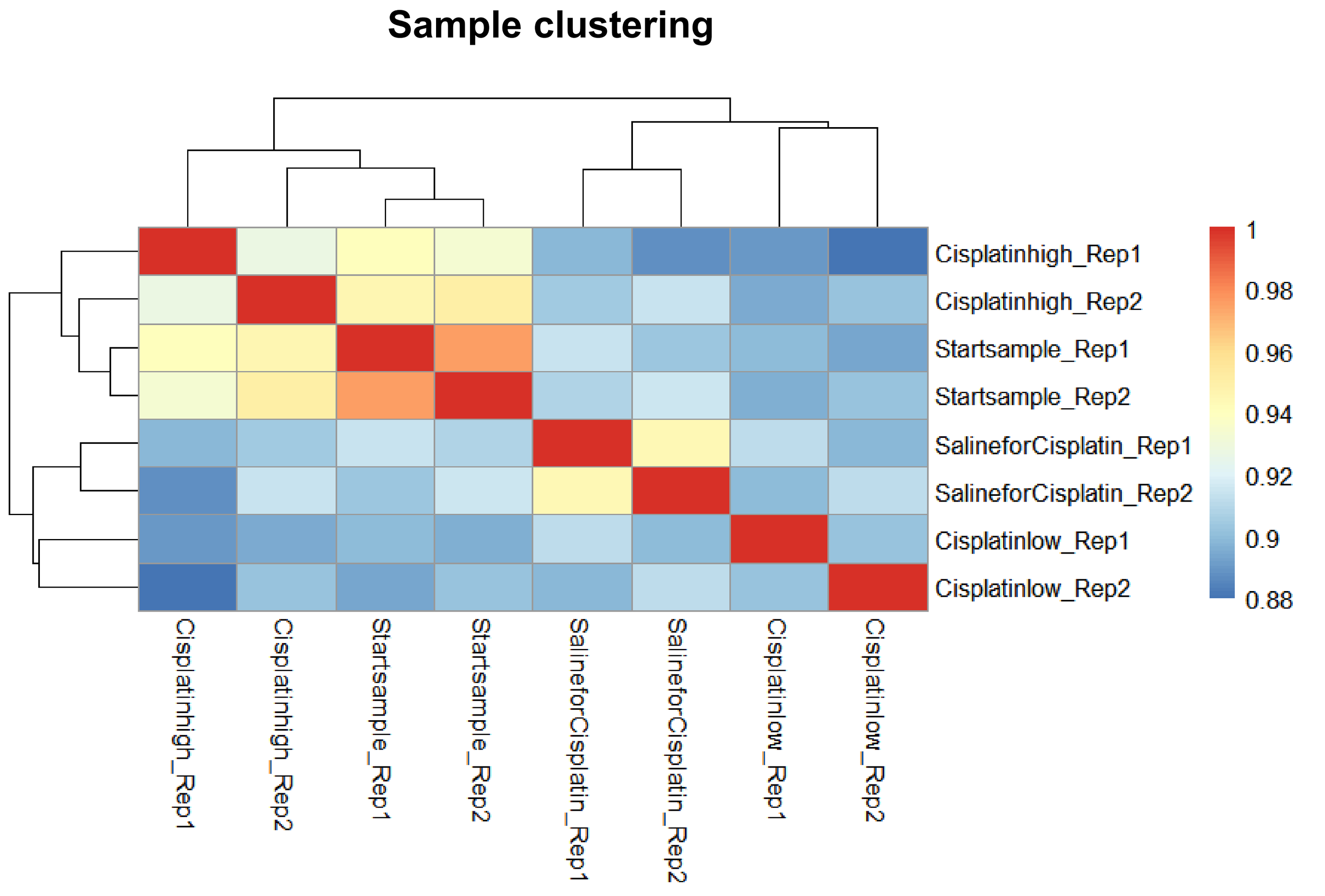

(B)

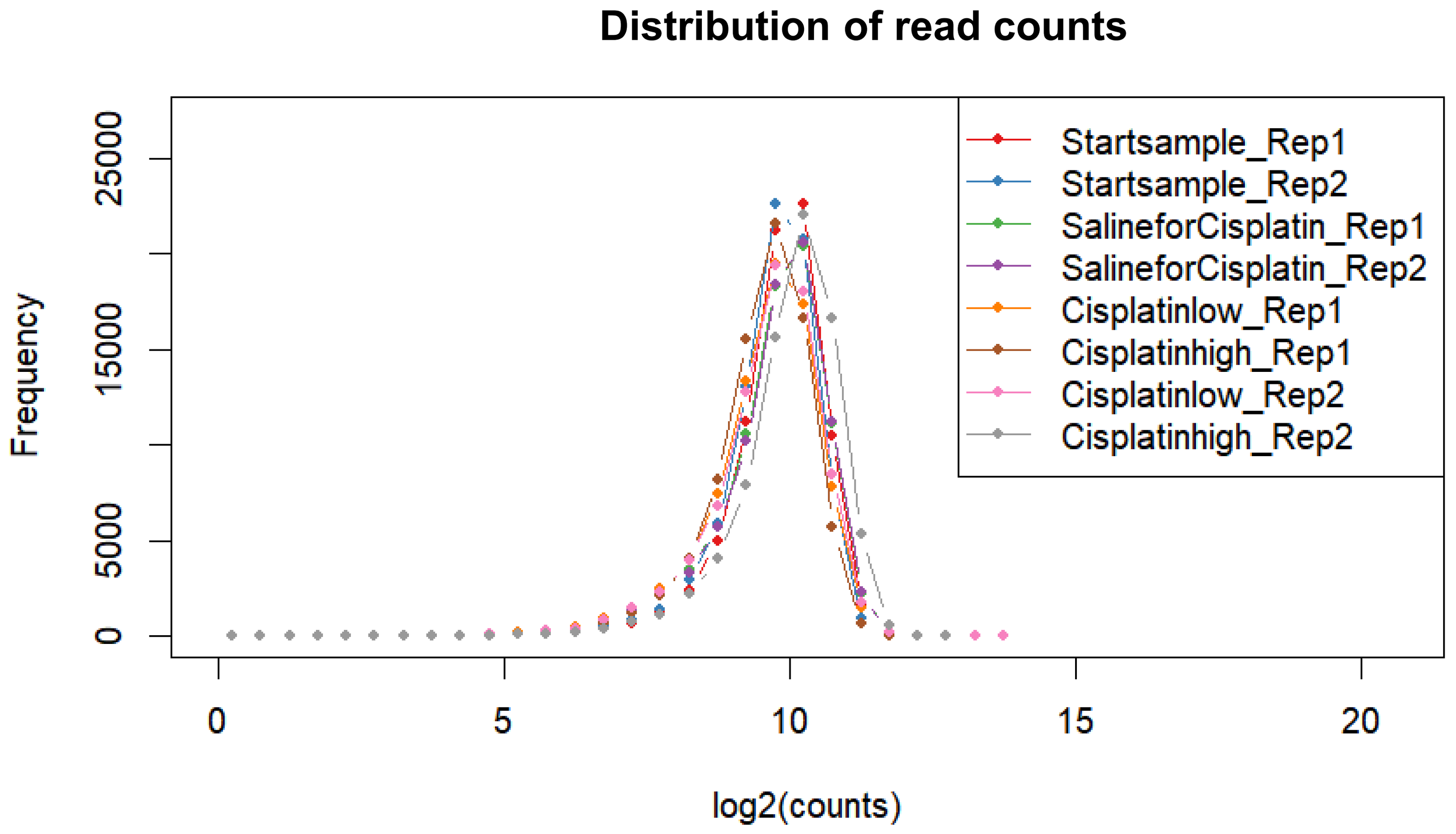

(E)

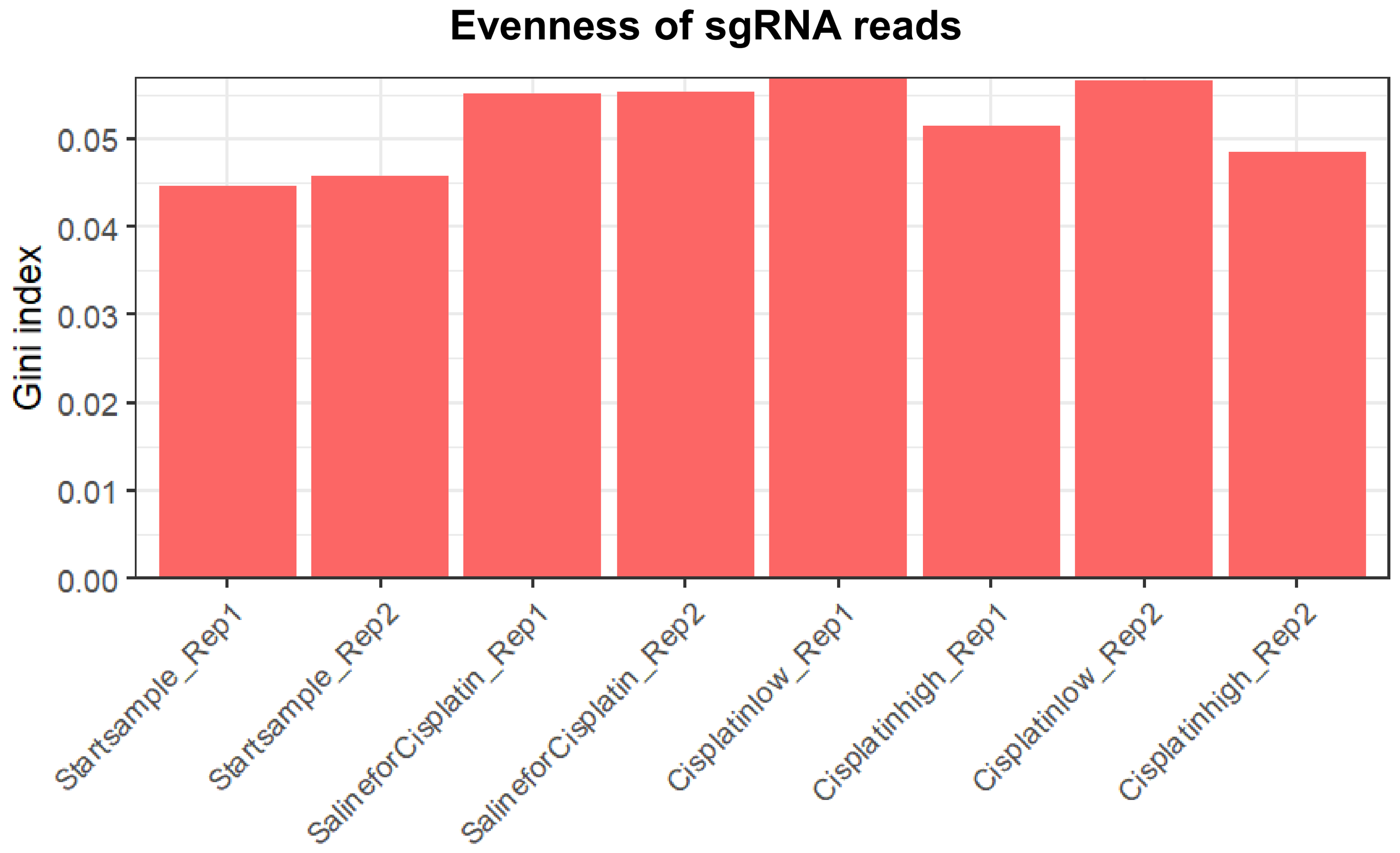

(C)

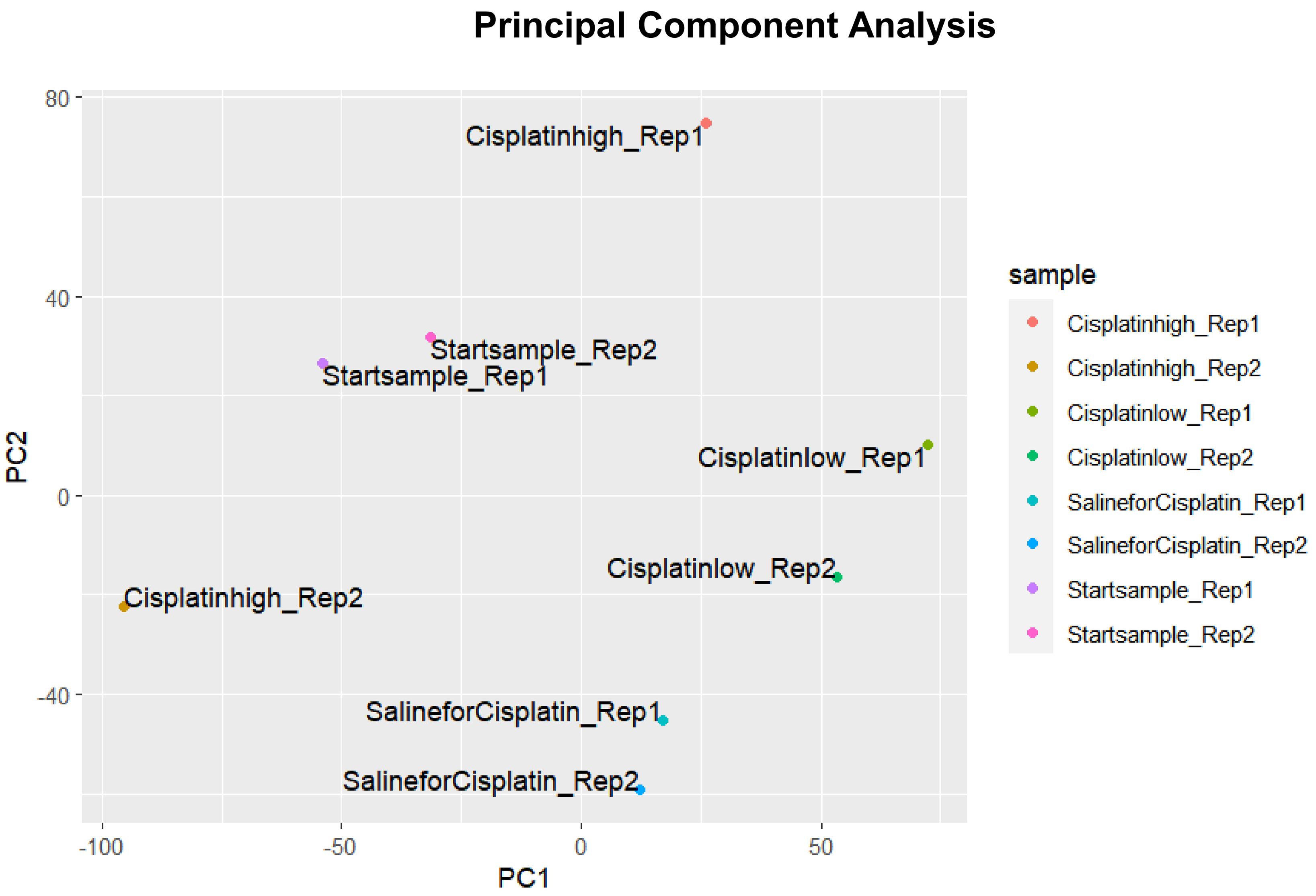

(F)

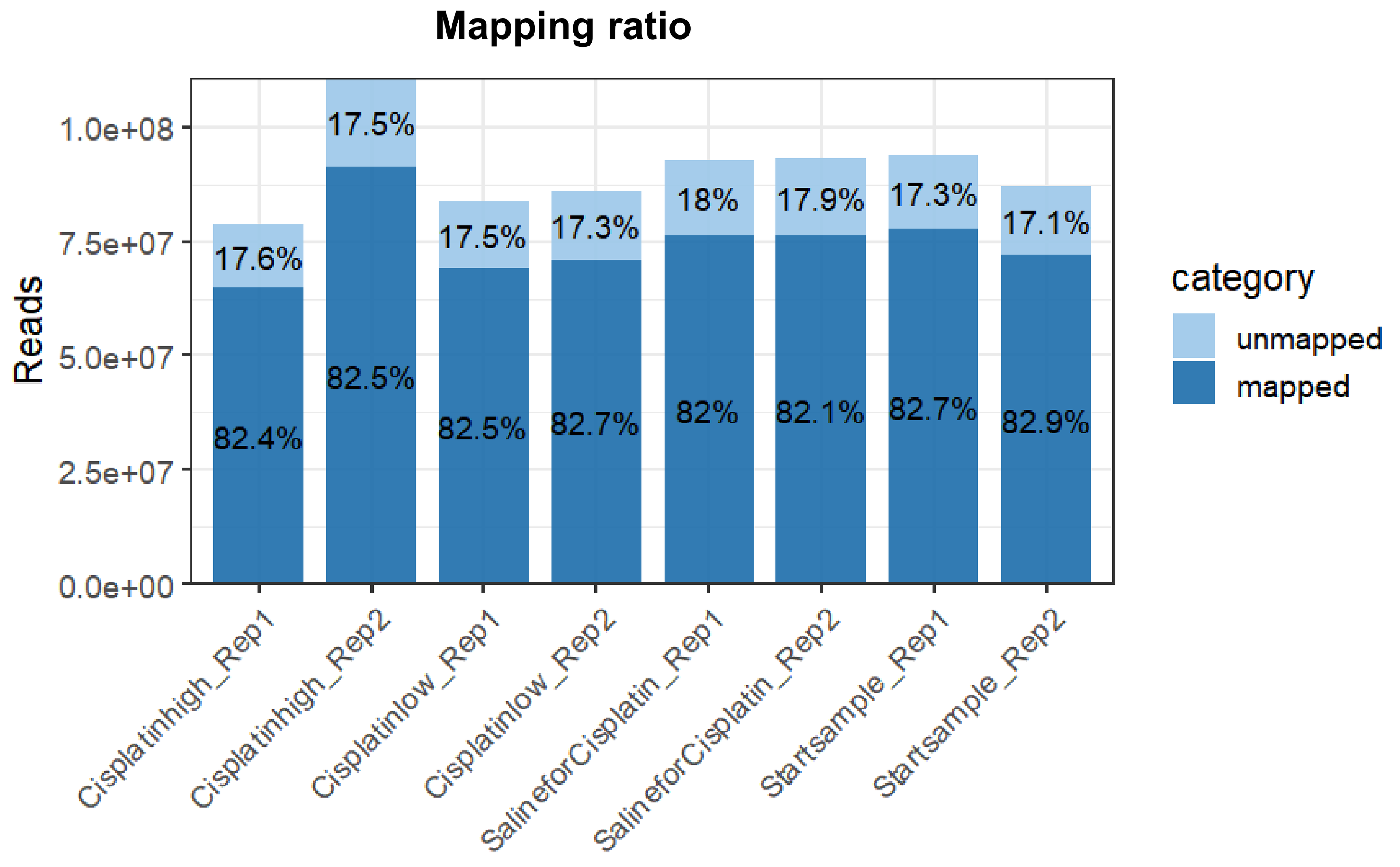

(G)

Label	Reads	Mapped	Percentage	Total gRNAs	Zerocounts	GiniIndex
Startsample_Rep1	94023063	77744860	0.8269	77441	28	0.04456
Startsample_Rep2	87008878	72115575	0.8288	77441	30	0.04572
SalineforCisplatin_Rep1	92943246	76223547	0.8201	77441	49	0.05521
SalineforCisplatin_Rep2	93143862	76464265	0.8209	77441	46	0.05529
Cisplatinlow_Rep1	83695888	69076722	0.8253	77441	45	0.05702
Cisplatinhigh_Rep1	78890138	64972695	0.8236	77441	63	0.05144
Cisplatinlow_Rep2	85995677	71102237	0.8268	77441	50	0.05662
Cisplatinhigh_Rep2	110778348	91411905	0.8252	77441	47	0.04850

Figure S2

(A)

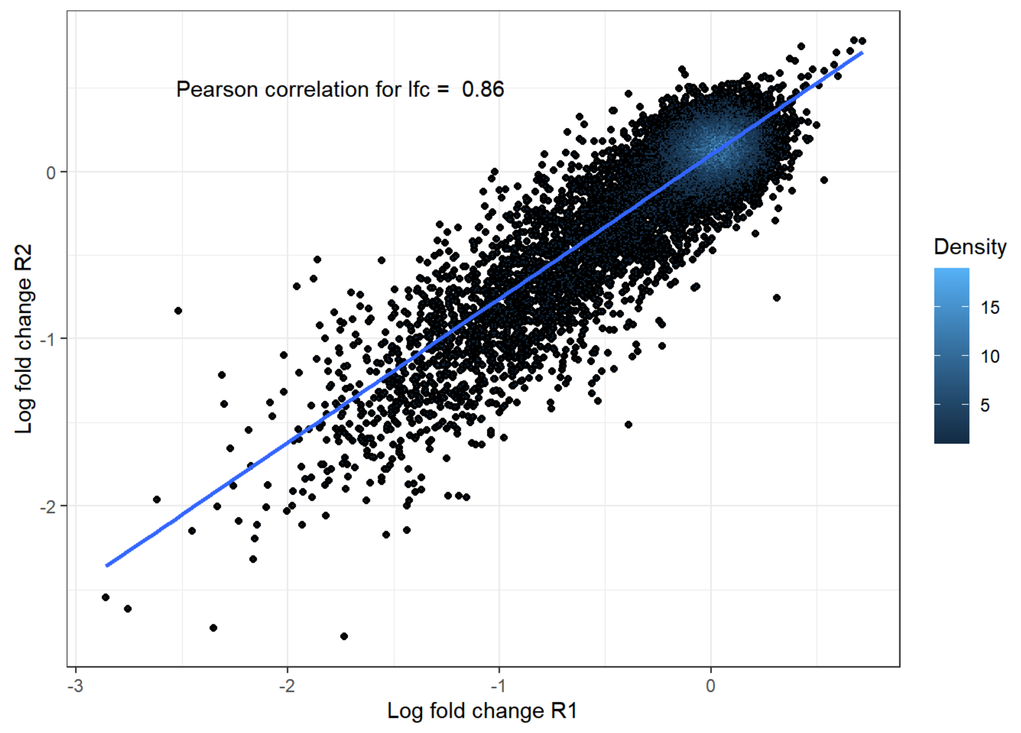

Replicate 1

Replicate 2

(B)

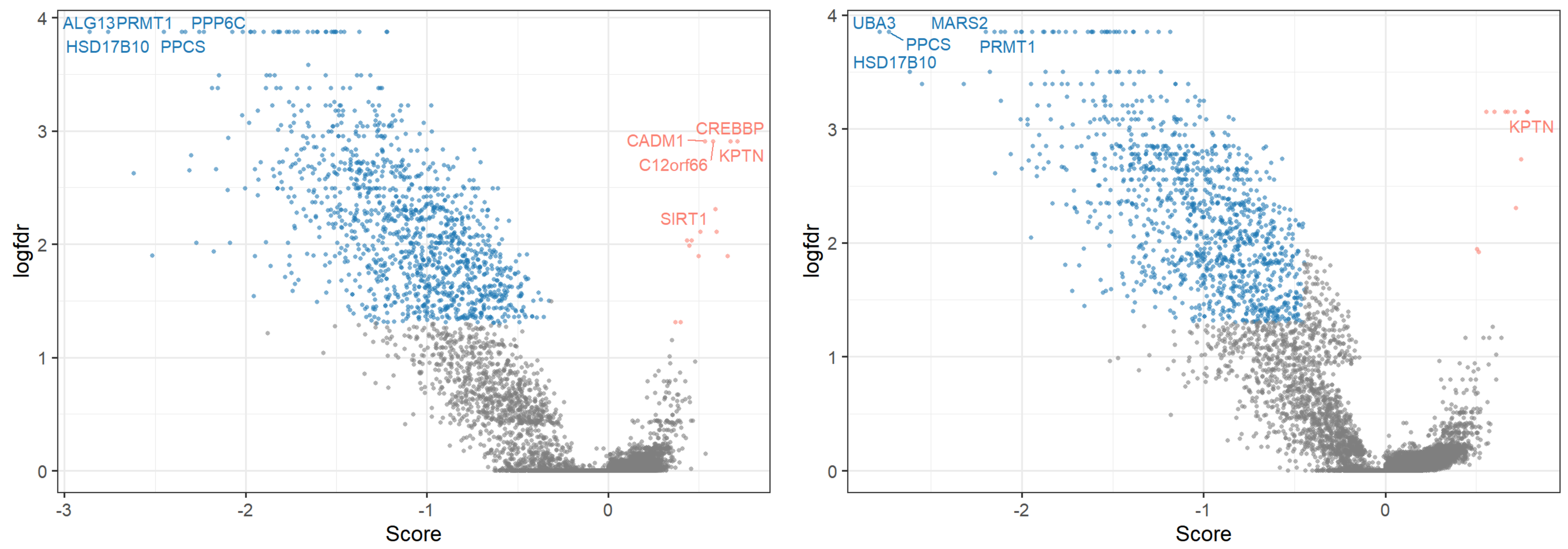

(C)

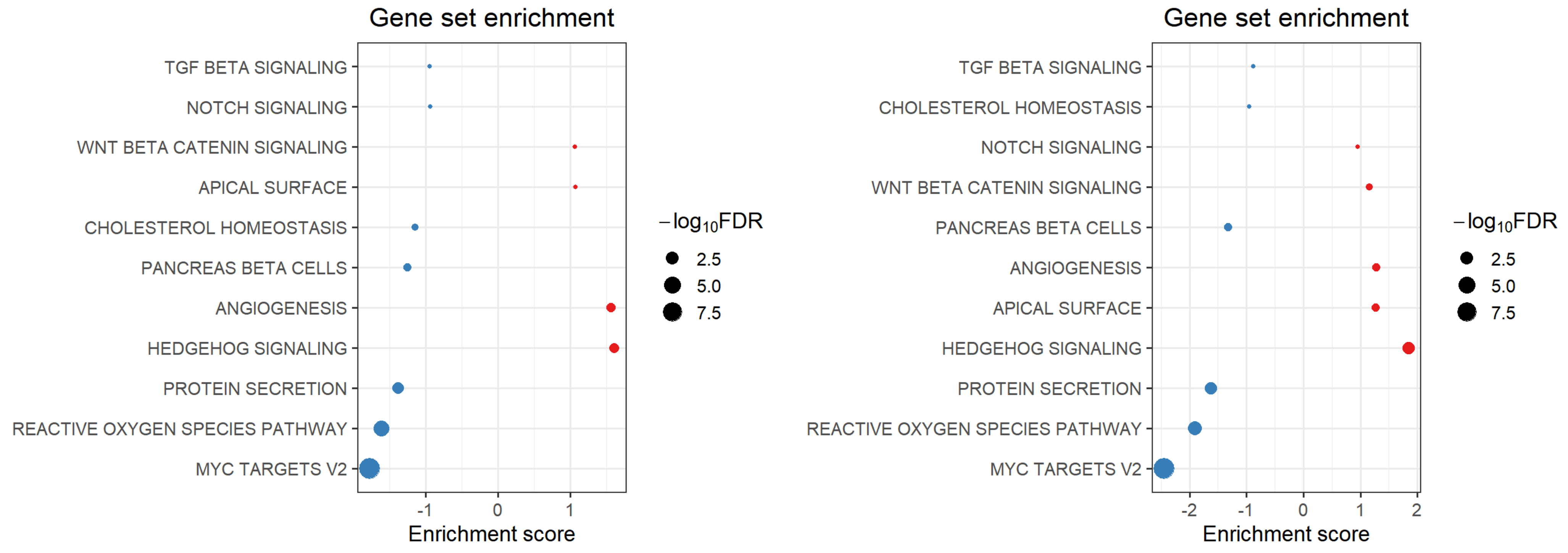

(D)

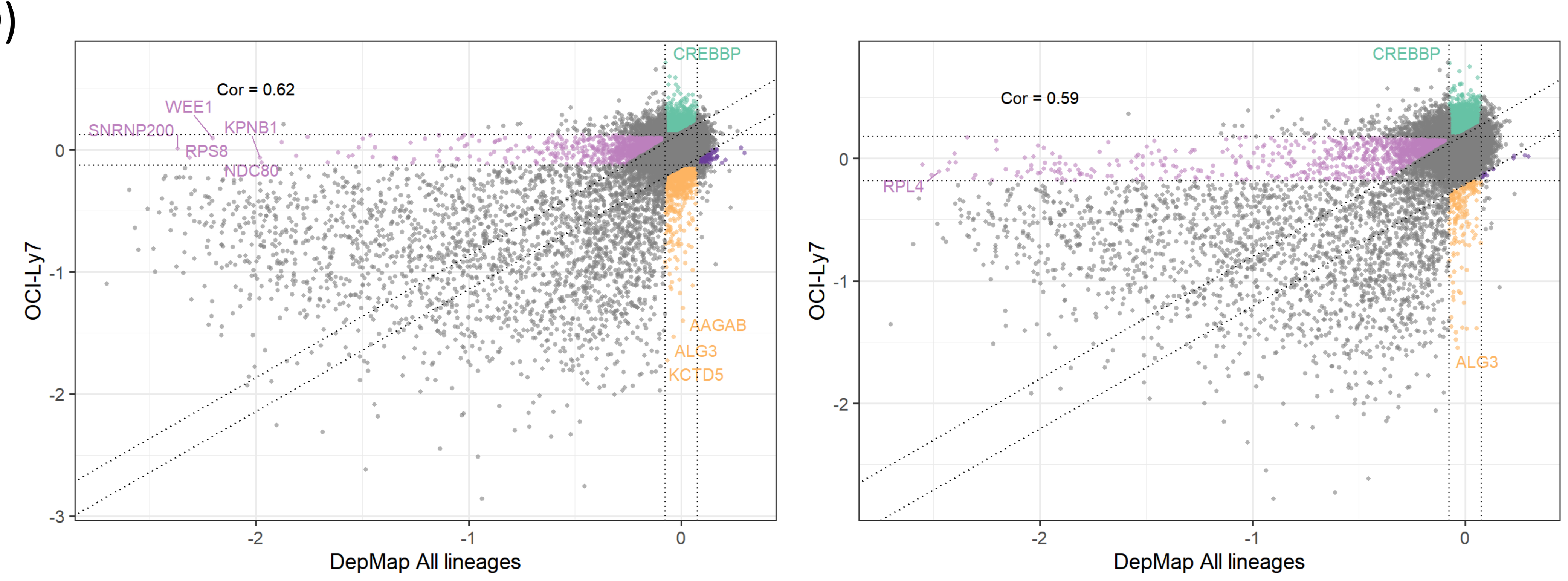

	DepMap_Lower	DepMap_Middle	DepMap_Top
R1_Lower	2174	526	2
R1_Middle	1448	11990	35
R1_Top	42	388	1

	DepMap_Lower	DepMap_Middle	DepMap_Top
R2_Lower	1673	188	1
R2_Middle	1948	12157	36
R2_Top	43	559	1

(E)

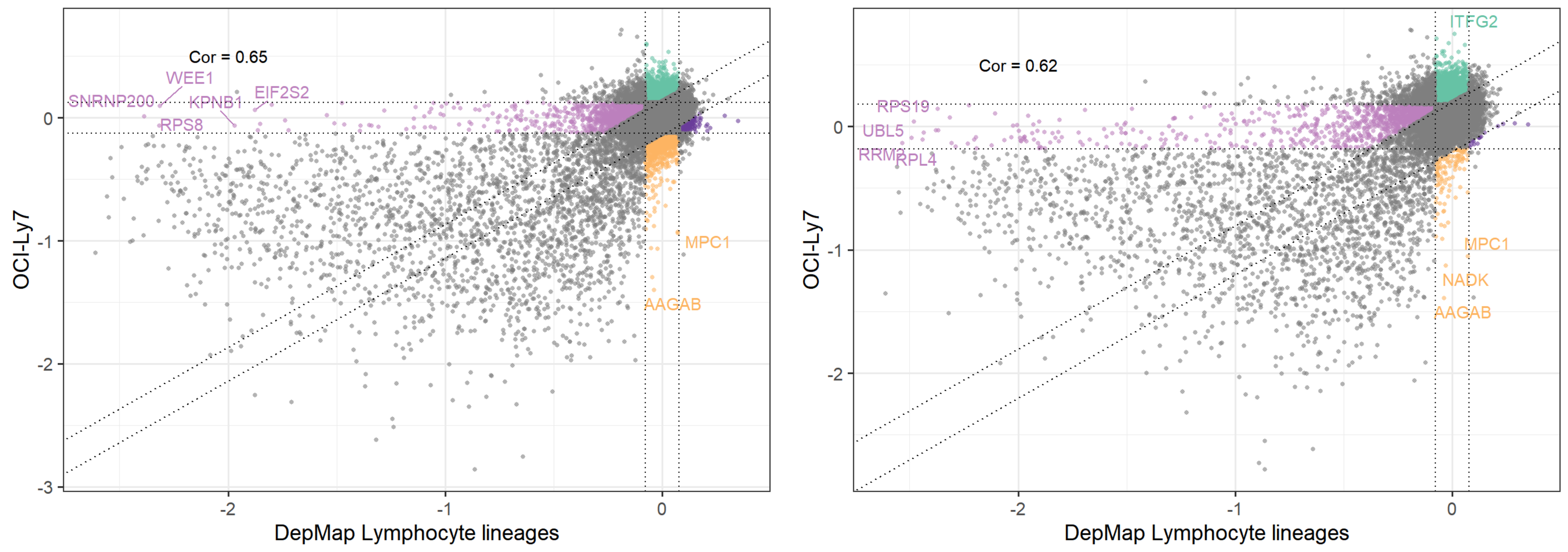

	DepMap_Lower	DepMap_Middle	DepMap_Top
R1_Lower	2251	451	0
R1_Middle	1245	12173	55
R1_Top	34	395	2

	DepMap_Lower	DepMap_Middle	DepMap_Top
R2_Lower	1740	122	0
R2_Middle	1748	12342	51
R2_Top	42	555	6

(F)

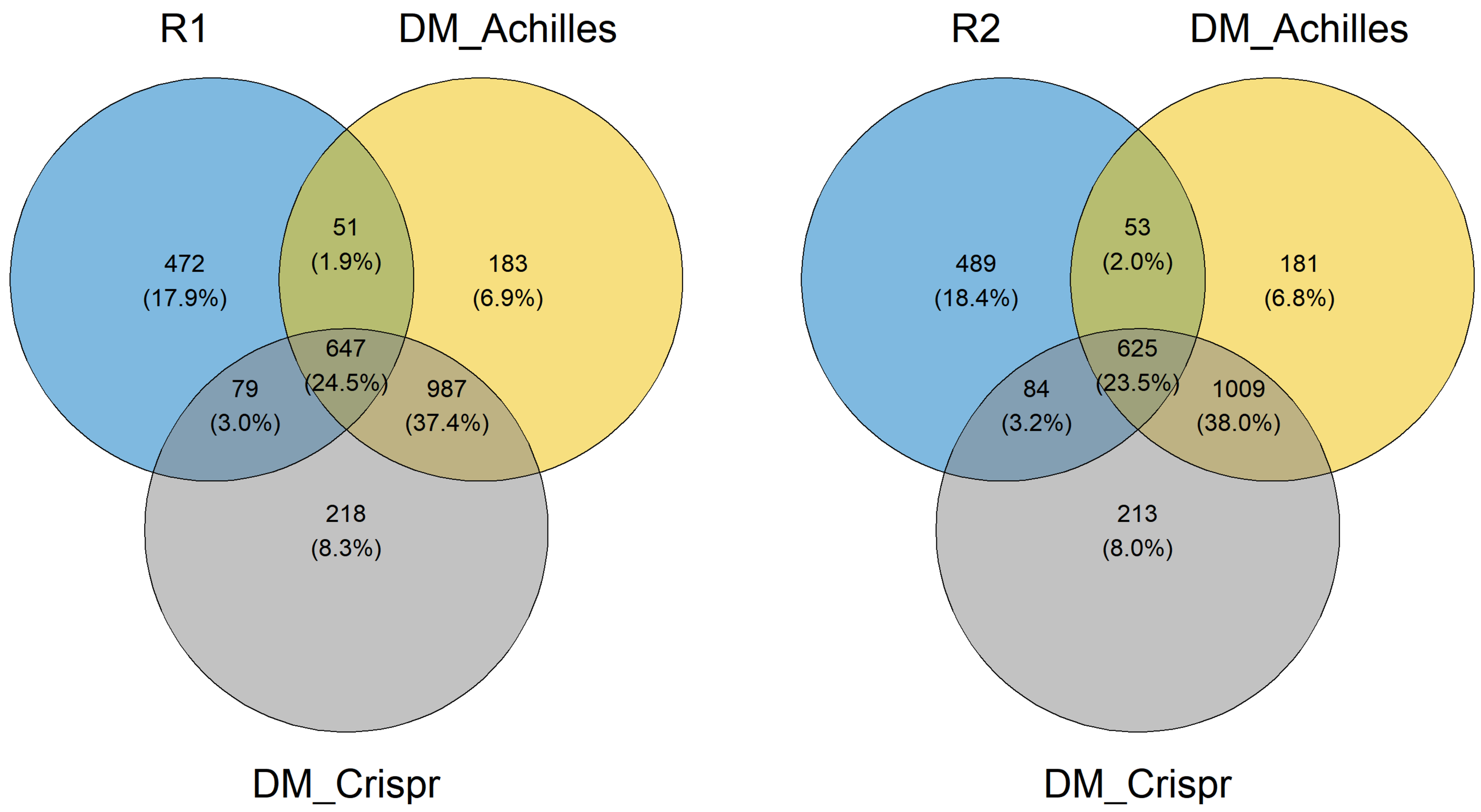

Figure S3

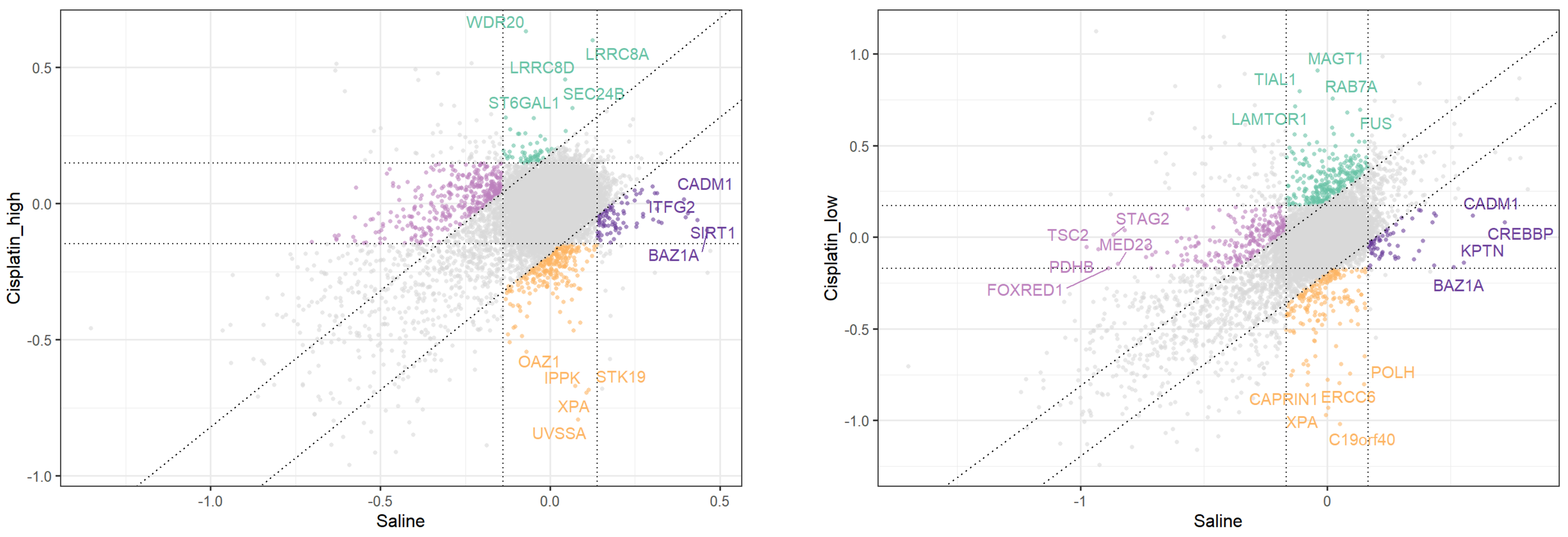

WDR20
LRRC8A
LRRC8D
SEC24B
ST6GAL1
CADM1
ITFG2
SIRT1
BAZ1A
OAZ1
IPPK
STK19
XPA
UVSSA
Cisplatin_high
Saline
MAGT1
TIAL1
RAB7A
LAMTOR1
FUS
STAG2
TSC2
MED23
PDHB
FOXRED1
CADM1
CREBBP
KPTN
BAZ1A
POLH
CAPRIN1
ERCC5
XPA
C19orf40
Cisplatin_low
Saline

Figure S4

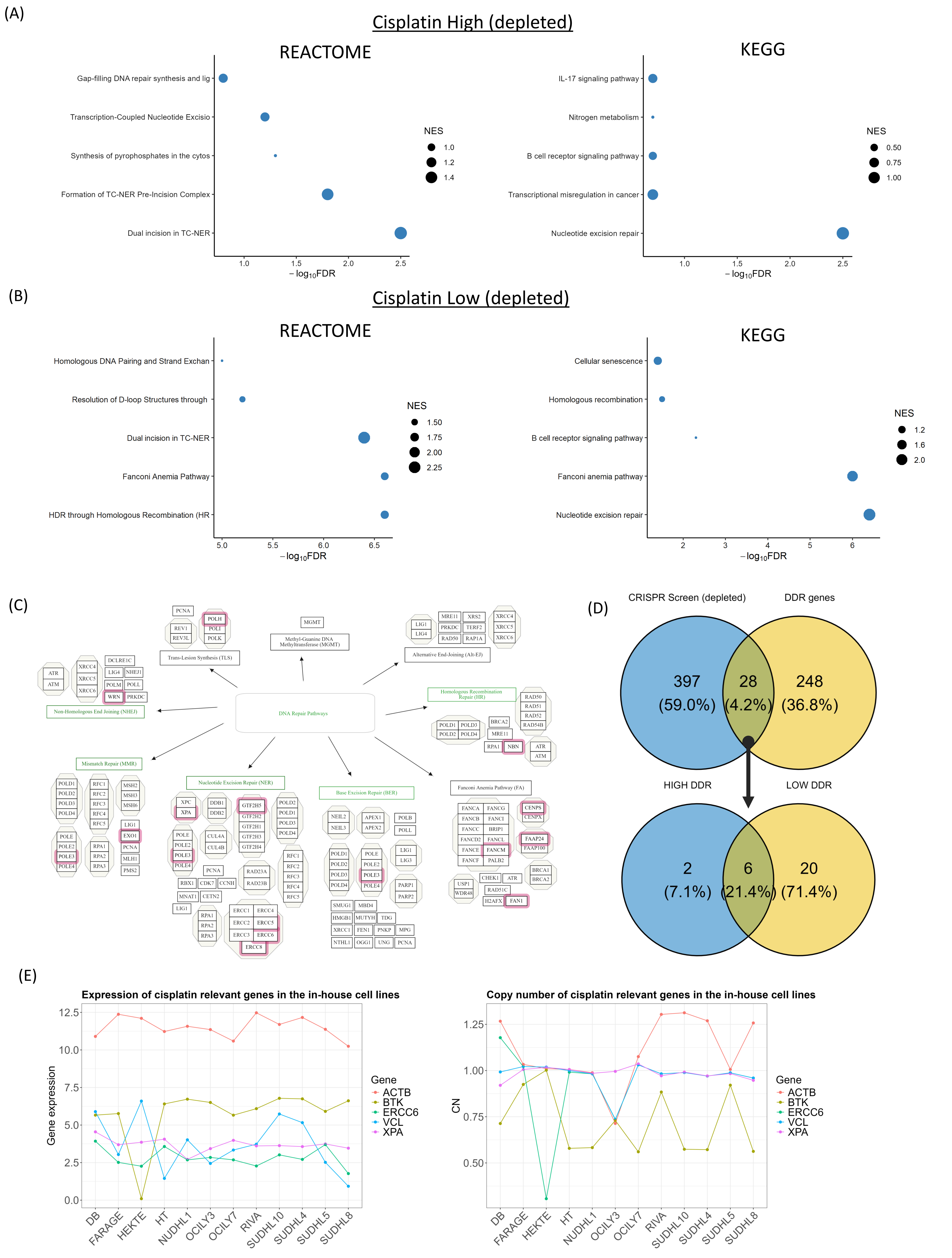
(A)
Cisplatin High (depleted)
REACTOME
Gap-filling DNA repair synthesis and lig
Transcription-Coupled Nucleotide Excisio
Synthesis of pyrophosphates in the cytos
Formation of TC-NER Pre-Incision Complex
Dual incision in TC-NER
NES
1.0
1.2
1.4
1.0 1.5 2.0 2.5
−log10FDR
KEGG
IL-17 signaling pathway
Nitrogen metabolism
B cell receptor signaling pathway
Transcriptional misregulation in cancer
Nucleotide excision repair
NES
0.50
0.75
1.00
1.0 1.5 2.0 2.5
−log10FDR
(B)
Cisplatin Low (depleted)
REACTOME
Homologous DNA Pairing and Strand Exchan
Resolution of D-loop Structures through
Dual incision in TC-NER
Fanconi Anemia Pathway
HDR through Homologous Recombination (HR
NES
1.50
1.75
2.00
2.25
5.0 5.5 6.0 6.5
−log10FDR
KEGG
Cellular senescence
Homologous recombination
B cell receptor signaling pathway
Fanconi anemia pathway
Nucleotide excision repair
NES
1.2
1.6
2.0
2 3 4 5 6
−log10FDR
(C)
DNA Repair Pathways
Trans-Lesion Synthesis (TLS)
Methyl-Guanine DNA Methyltransferase (MGMT)
Alternative End-Joining (Alt-EJ)
Non-Homologous End Joining (NHEJ)
Homologous Recombination Repair (HR)
Mismatch Repair (MMR)
Nucleotide Excision Repair (NER)
Base Excision Repair (BER)
Fanconi Anemia Pathway (FA)
(D)
CRISPR Screen (depleted)
DDR genes
397 (59.0%)
28 (4.2%)
248 (36.8%)
HIGH DDR
LOW DDR
2 (7.1%)
6 (21.4%)
20 (71.4%)
(E)
Expression of cisplatin relevant genes in the in-house cell lines
Gene expression
12.5 10.0 7.5 5.0 2.5 0.0
Gene
ACTB
BTK
ERCC6
VCL
XPA
DB FARAGE HEKTE HT NUDHL1 OCILY3 OCILY7 RIVA SUDHL10 SUDHL4 SUDHL5 SUDHL8
Copy number of cisplatin relevant genes in the in-house cell lines
CN
1.25 1.00 0.75 0.50
Gene
ACTB
BTK
ERCC6
VCL
XPA
DB FARAGE HEKTE HT NUDHL1 OCILY3 OCILY7 RIVA SUDHL10 SUDHL4 SUDHL5 SUDHL8

Figure S5

(A)

HBL1

IBR \ CIS	0 µg/mL	0.43 µg/mL	0.85 µg/mL	1.7 µg/mL	3.4 µg/mL	6.8 µg/mL
0 µM	1.00	1.00	0.86	0.49	0.23	0.11
0.25 µM	0.86	0.8 (Bliss: -0.06)	0.72 (Bliss: -0.02)	0.42 (Bliss: 0.01)	0.19 (Bliss: -0.01)	0.09 (Bliss: -0.01)
1 µM	0.82	0.81 (Bliss: -0.01)	0.73 (Bliss: 0.02)	0.45 (Bliss: 0.06)	0.24 (Bliss: 0.05)	0.12 (Bliss: 0.03)
4 µM	0.76	0.76 (Bliss: -0.01)	0.71 (Bliss: 0.05)	0.53 (Bliss: 0.16)	0.32 (Bliss: 0.15)	0.16 (Bliss: 0.07)
16 µM	0.61	0.58 (Bliss: -0.02)	0.56 (Bliss: 0.04)	0.46 (Bliss: 0.16)	0.32 (Bliss: 0.18)	0.16 (Bliss: 0.09)
32 µM	0.25	0.26 (Bliss: 0.01)	0.25 (Bliss: 0.04)	0.22 (Bliss: 0.1)	0.15 (Bliss: 0.09)	0.07 (Bliss: 0.05)

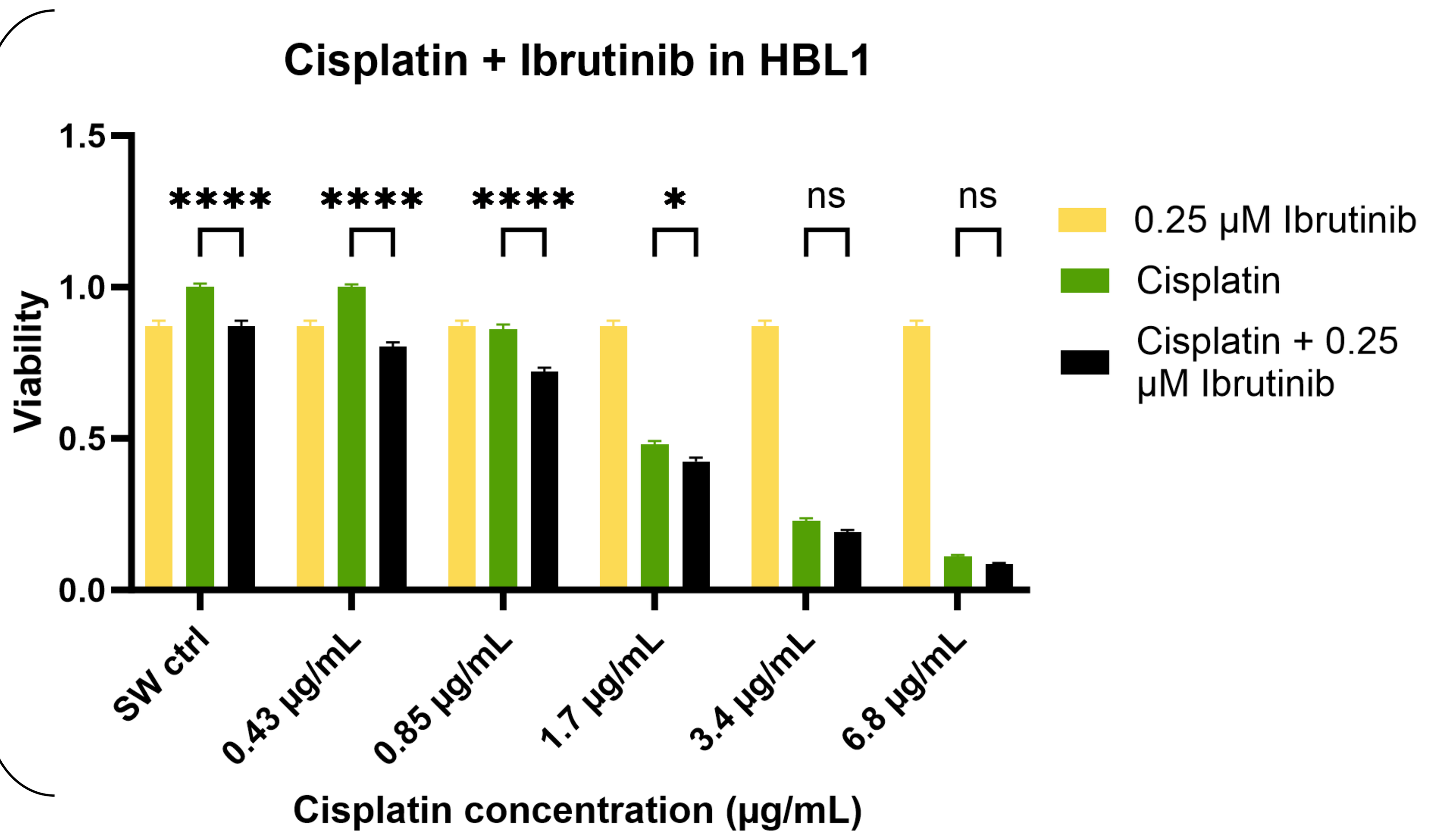

OCILY7

IBR \ CIS	0 µg/mL	0.43 µg/mL	0.85 µg/mL	1.7 µg/mL	3.4 µg/mL	6.8 µg/mL
0 µM	1.00	0.91	0.95	0.84	0.86	0.60
0.25 µM	0.98	0.8 (Bliss: -0.1)	0.73 (Bliss: -0.2)	0.74 (Bliss: -0.08)	0.66 (Bliss: -0.18)	0.43 (Bliss: -0.16)
1 µM	0.90	0.74 (Bliss: -0.09)	0.66 (Bliss: -0.19)	0.71 (Bliss: -0.05)	0.66 (Bliss: -0.12)	0.44 (Bliss: -0.1)
4 µM	0.62	0.55 (Bliss: -0.01)	0.49 (Bliss: -0.09)	0.52 (Bliss: 0)	0.47 (Bliss: -0.06)	0.36 (Bliss: 0)
16 µM	0.36	0.34 (Bliss: 0.01)	0.34 (Bliss: -0.01)	0.4 (Bliss: 0.1)	0.35 (Bliss: 0.04)	0.27 (Bliss: 0.05)
32 µM	0.09	0.14 (Bliss: 0.05)	0.13 (Bliss: 0.04)	0.14 (Bliss: 0.06)	0.08 (Bliss: 0)	0.07 (Bliss: 0.01)

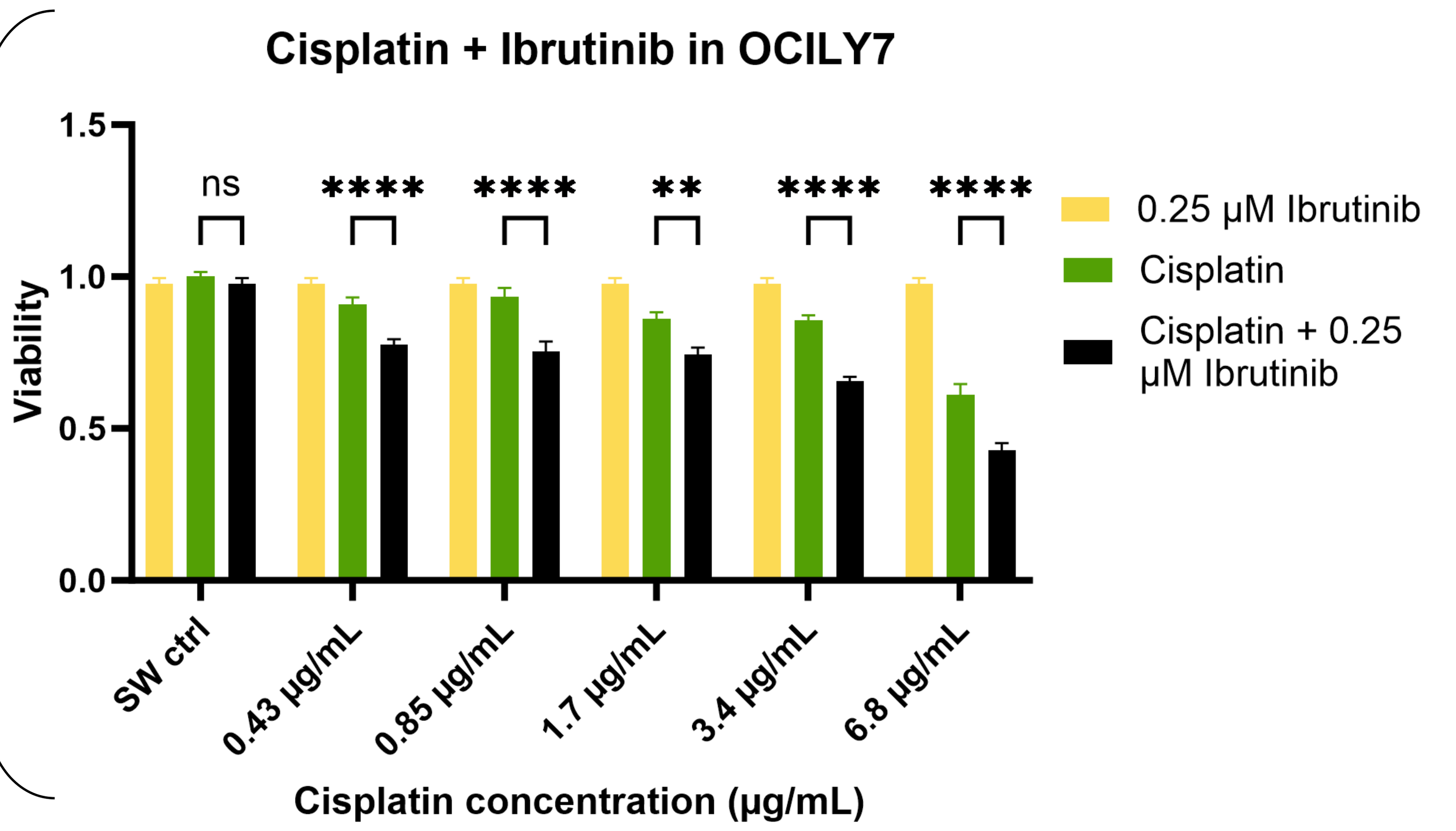

RIVA

IBR \ CIS	0 µg/mL	0.45 µg/mL	0.85 µg/mL	1.7 µg/mL	3.4 µg/mL	6.8 µg/mL
0 µM	1.00	0.99	0.98	0.92	0.80	0.44
0.25 µM	0.93	0.87 (Bliss: -0.04)	0.84 (Bliss: -0.07)	0.8 (Bliss: -0.06)	0.65 (Bliss: -0.09)	0.36 (Bliss: -0.04)
1 µM	0.83	0.8 (Bliss: -0.02)	0.77 (Bliss: -0.04)	0.76 (Bliss: -0.01)	0.65 (Bliss: -0.02)	0.43 (Bliss: 0.07)
4 µM	0.74	0.74 (Bliss: 0)	0.71 (Bliss: -0.02)	0.7 (Bliss: 0.02)	0.61 (Bliss: 0.02)	0.5 (Bliss: 0.18)
16 µM	0.66	0.65 (Bliss: 0)	0.65 (Bliss: 0)	0.62 (Bliss: 0.01)	0.59 (Bliss: 0.06)	0.55 (Bliss: 0.26)
32 µM	0.39	0.36 (Bliss: -0.02)	0.36 (Bliss: -0.02)	0.29 (Bliss: -0.07)	0.36 (Bliss: 0.05)	0.3 (Bliss: 0.13)

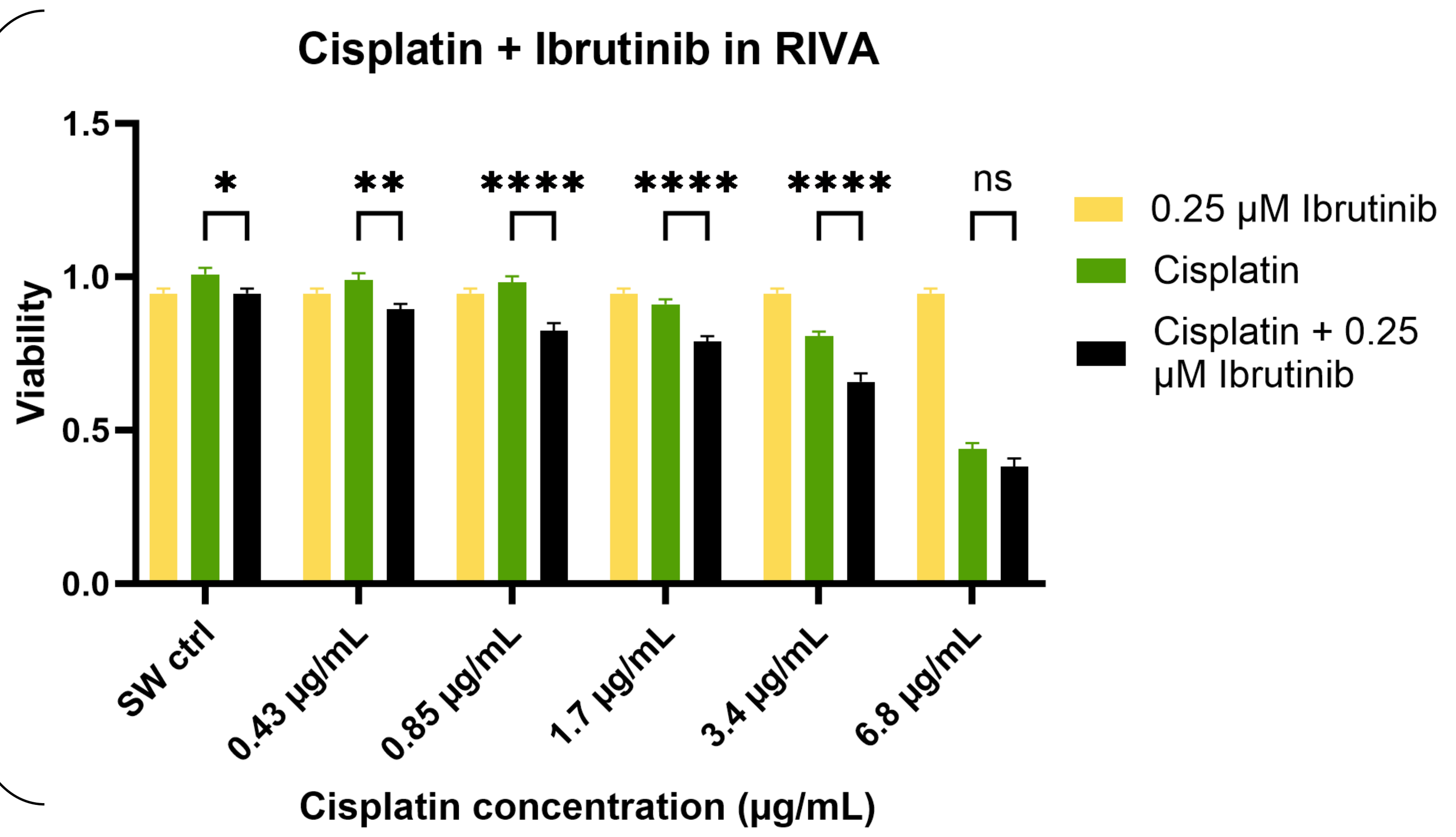

SUDHL5

IBR \ CIS	0 µg/mL	0.2125 µg/mL	0.425 µg/mL	0.85 µg/mL	1.7 µg/mL	3.4 µg/mL
0 µM	1.00	1.03	0.91	0.58	0.17	0.06
0.25 µM	0.93	0.78 (Bliss: -0.17)	0.74 (Bliss: -0.11)	0.46 (Bliss: -0.08)	0.08 (Bliss: -0.08)	0.01 (Bliss: -0.05)
1 µM	0.86	0.79 (Bliss: -0.09)	0.73 (Bliss: -0.05)	0.51 (Bliss: 0.01)	0.13 (Bliss: -0.01)	0.05 (Bliss: -0.01)
4 µM	0.72	0.66 (Bliss: -0.08)	0.63 (Bliss: -0.03)	0.45 (Bliss: 0.03)	0.15 (Bliss: 0.02)	0.04 (Bliss: -0.01)
16 µM	0.40	0.42 (Bliss: 0)	0.36 (Bliss: -0.01)	0.24 (Bliss: 0)	0.09 (Bliss: 0.02)	0.04 (Bliss: 0.01)
32 µM	0.03	0.04 (Bliss: 0.01)	0.04 (Bliss: 0.01)	0.03 (Bliss: 0.01)	0.03 (Bliss: 0.03)	0.02 (Bliss: 0.02)

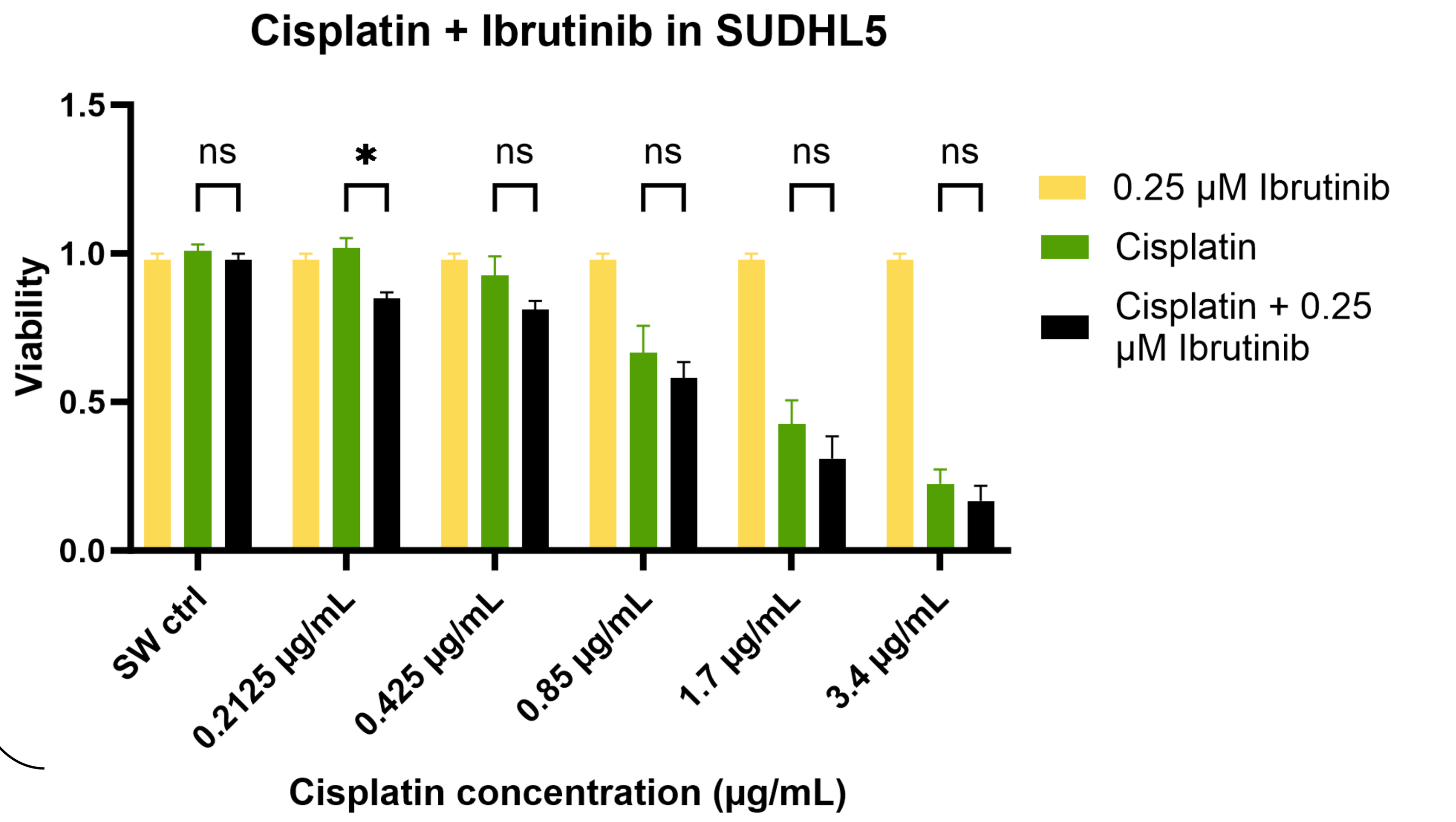

Figure S5

(B)

HBL1

IBR \ CAR	0 µg/mL	5 µg/mL	10 µg/mL	30 µg/mL	50 µg/mL	70 µg/mL
0 µM	1.00	1.02	0.94	0.38	0.21	0.14
0.25 µM	0.88	0.8 (Bliss: -0.1)	0.71 (Bliss: -0.12)	0.31 (Bliss: -0.03)	0.15 (Bliss: -0.03)	0.09 (Bliss: -0.03)
1 µM	0.86	0.79 (Bliss: -0.09)	0.72 (Bliss: -0.08)	0.33 (Bliss: 0.01)	0.18 (Bliss: 0)	0.13 (Bliss: 0.01)
4 µM	0.80	0.72 (Bliss: -0.09)	0.65 (Bliss: -0.1)	0.37 (Bliss: 0.06)	0.23 (Bliss: 0.06)	0.17 (Bliss: 0.06)
16 µM	0.61	0.56 (Bliss: -0.06)	0.52 (Bliss: -0.06)	0.35 (Bliss: 0.12)	0.22 (Bliss: 0.1)	0.14 (Bliss: 0.06)
32 µM	0.30	0.29 (Bliss: -0.02)	0.23 (Bliss: -0.05)	0.15 (Bliss: 0.04)	0.08 (Bliss: 0.02)	0.06 (Bliss: 0.01)

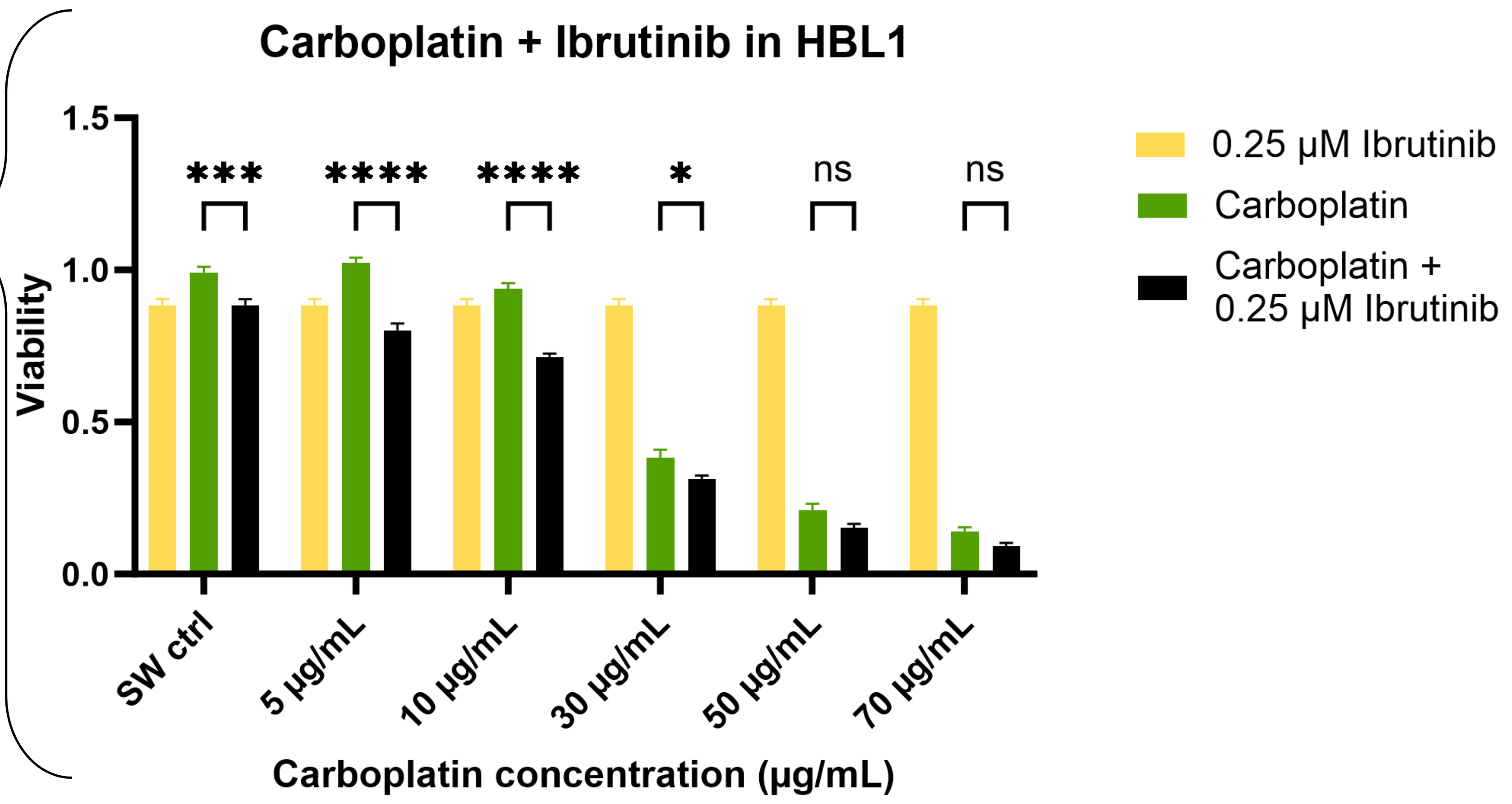

OCILY7

IBR \ CAR	0 µg/mL	5 µg/mL	10 µg/mL	30 µg/mL	50 µg/mL	70 µg/mL
0 µM	1.00	1.00	1.09	1.00	0.87	0.57
0.25 µM	0.98	0.74 (Bliss: -0.24)	0.73 (Bliss: -0.33)	0.76 (Bliss: -0.21)	0.66 (Bliss: -0.19)	0.39 (Bliss: -0.16)
1 µM	0.96	0.72 (Bliss: -0.25)	0.71 (Bliss: -0.34)	0.75 (Bliss: -0.21)	0.65 (Bliss: -0.19)	0.38 (Bliss: -0.17)
4 µM	0.79	0.63 (Bliss: -0.17)	0.58 (Bliss: -0.28)	0.6 (Bliss: -0.19)	0.52 (Bliss: -0.17)	0.31 (Bliss: -0.14)
16 µM	0.58	0.51 (Bliss: -0.08)	0.48 (Bliss: -0.15)	0.45 (Bliss: -0.13)	0.39 (Bliss: -0.12)	0.25 (Bliss: -0.08)
32 µM	0.14	0.15 (Bliss: 0.02)	0.14 (Bliss: -0.01)	0.09 (Bliss: -0.04)	0.08 (Bliss: -0.04)	0.05 (Bliss: -0.02)

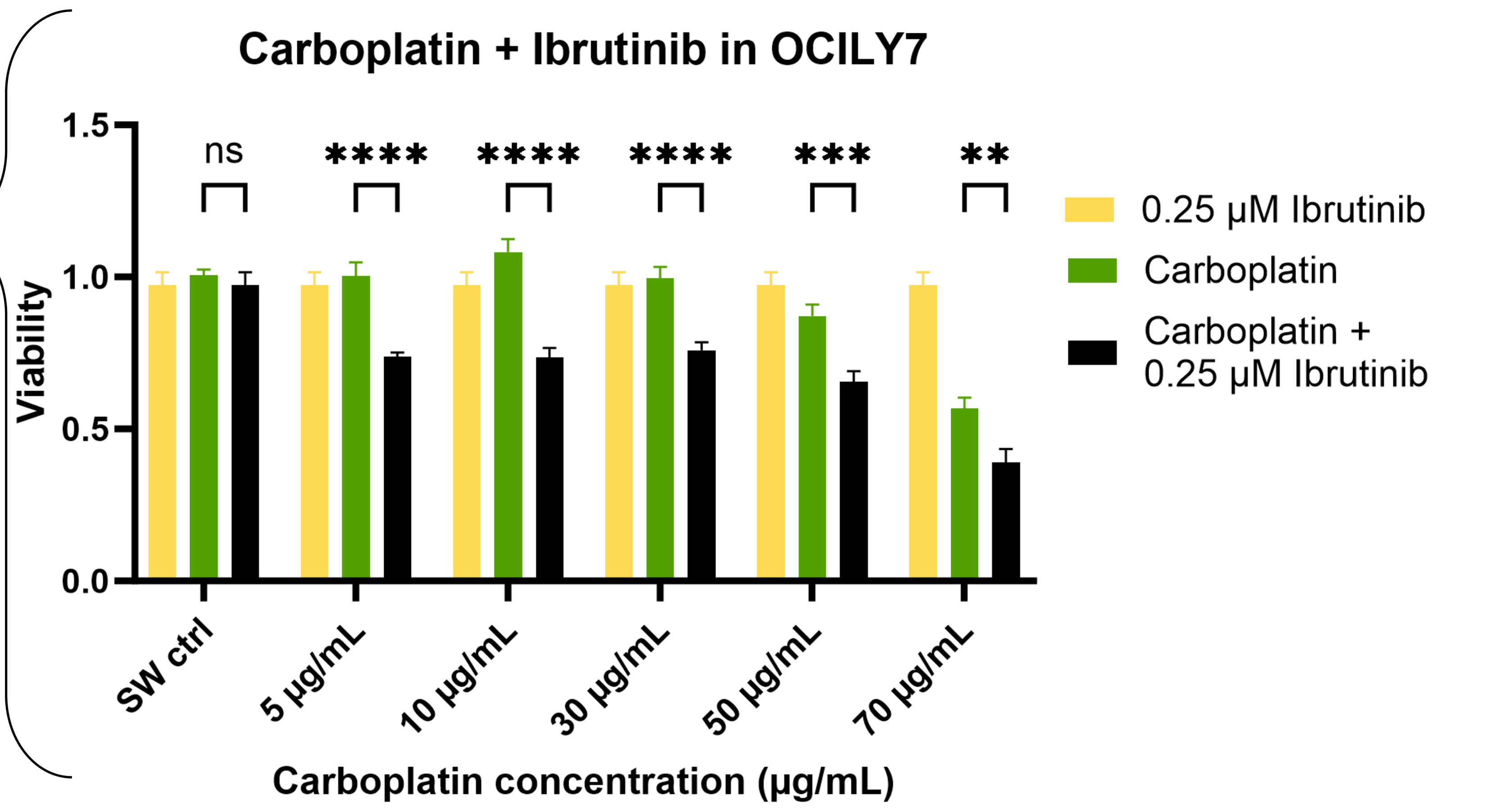

RIVA

IBR \ CAR	0 µg/mL	5 µg/mL	10 µg/mL	30 µg/mL	50 µg/mL	70 µg/mL
0 µM	1.00	1.03	1.02	0.90	0.72	0.47
0.25 µM	0.87	0.77 (Bliss: -0.12)	0.76 (Bliss: -0.13)	0.63 (Bliss: -0.15)	0.49 (Bliss: -0.13)	0.28 (Bliss: -0.12)
1 µM	0.79	0.7 (Bliss: -0.11)	0.7 (Bliss: -0.11)	0.62 (Bliss: -0.09)	0.51 (Bliss: -0.05)	0.37 (Bliss: 0)
4 µM	0.70	0.67 (Bliss: -0.06)	0.64 (Bliss: -0.07)	0.58 (Bliss: -0.05)	0.49 (Bliss: -0.01)	0.4 (Bliss: 0.07)
16 µM	0.61	0.59 (Bliss: -0.05)	0.58 (Bliss: -0.05)	0.55 (Bliss: -0.01)	0.48 (Bliss: 0.04)	0.41 (Bliss: 0.13)
32 µM	0.35	0.34 (Bliss: -0.02)	0.33 (Bliss: -0.03)	0.31 (Bliss: -0.01)	0.28 (Bliss: 0.02)	0.25 (Bliss: 0.08)

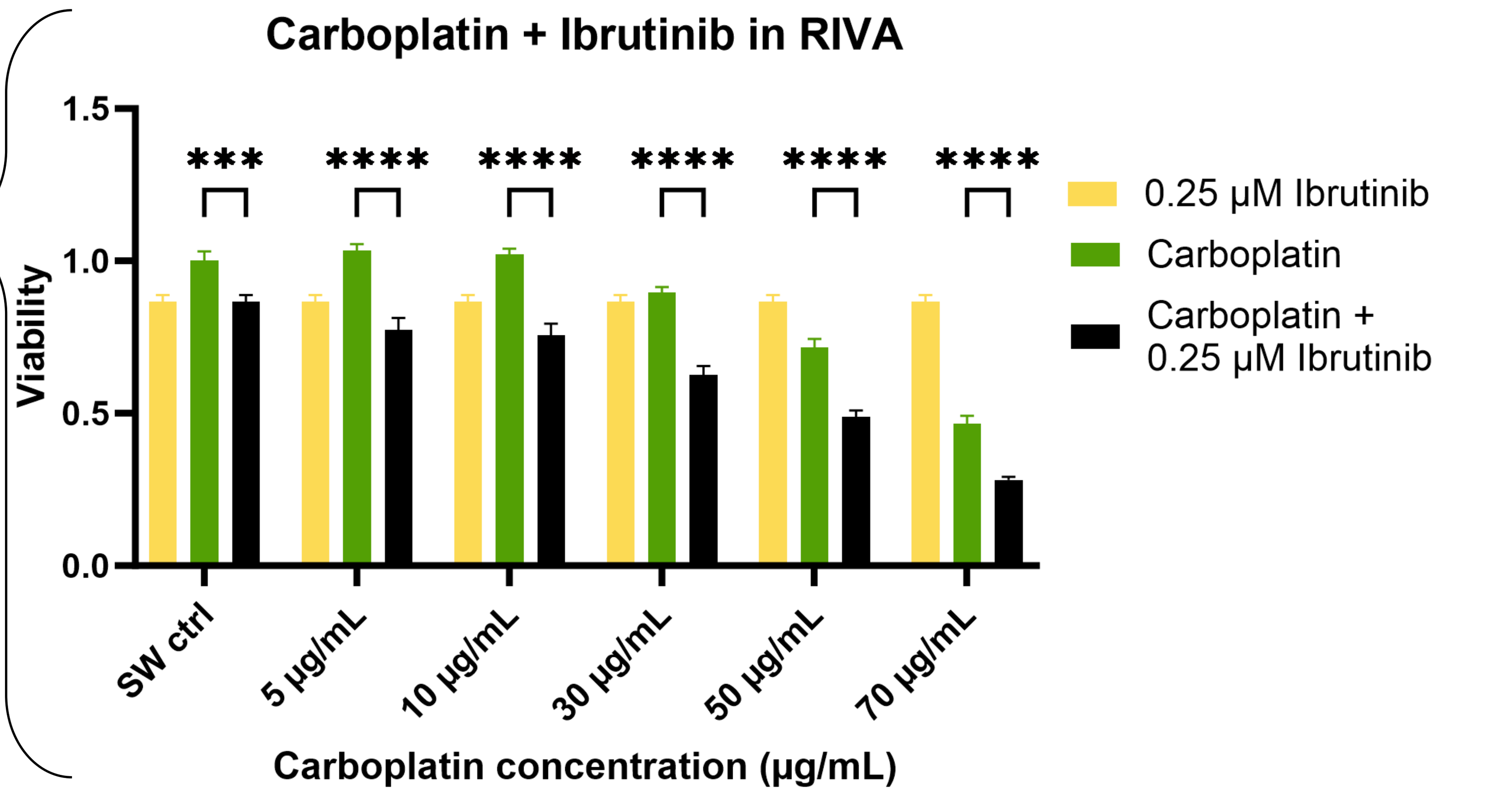

SUDHL5

IBR \ CAR	0 µg/mL	2.5 µg/mL	5 µg/mL	7.5 µg/mL	15 µg/mL	30 µg/mL
0 µM	1.00	0.85	0.69	0.39	0.13	0.08
0.25 µM	0.88	0.67 (Bliss: -0.08)	0.45 (Bliss: -0.15)	0.27 (Bliss: -0.07)	0.07 (Bliss: -0.04)	0.02 (Bliss: -0.05)
1 µM	0.83	0.69 (Bliss: -0.01)	0.5 (Bliss: -0.07)	0.3 (Bliss: -0.02)	0.08 (Bliss: -0.02)	0.06 (Bliss: 0)
4 µM	0.82	0.63 (Bliss: -0.07)	0.48 (Bliss: -0.09)	0.3 (Bliss: -0.03)	0.08 (Bliss: -0.03)	0.04 (Bliss: -0.02)
16 µM	0.35	0.27 (Bliss: -0.02)	0.18 (Bliss: -0.06)	0.11 (Bliss: -0.02)	0.05 (Bliss: 0.01)	0.03 (Bliss: 0.01)
32 µM	0.05	0.04 (Bliss: 0)	0.03 (Bliss: 0)	0.03 (Bliss: 0.01)	0.03 (Bliss: 0.02)	0.02 (Bliss: 0.02)

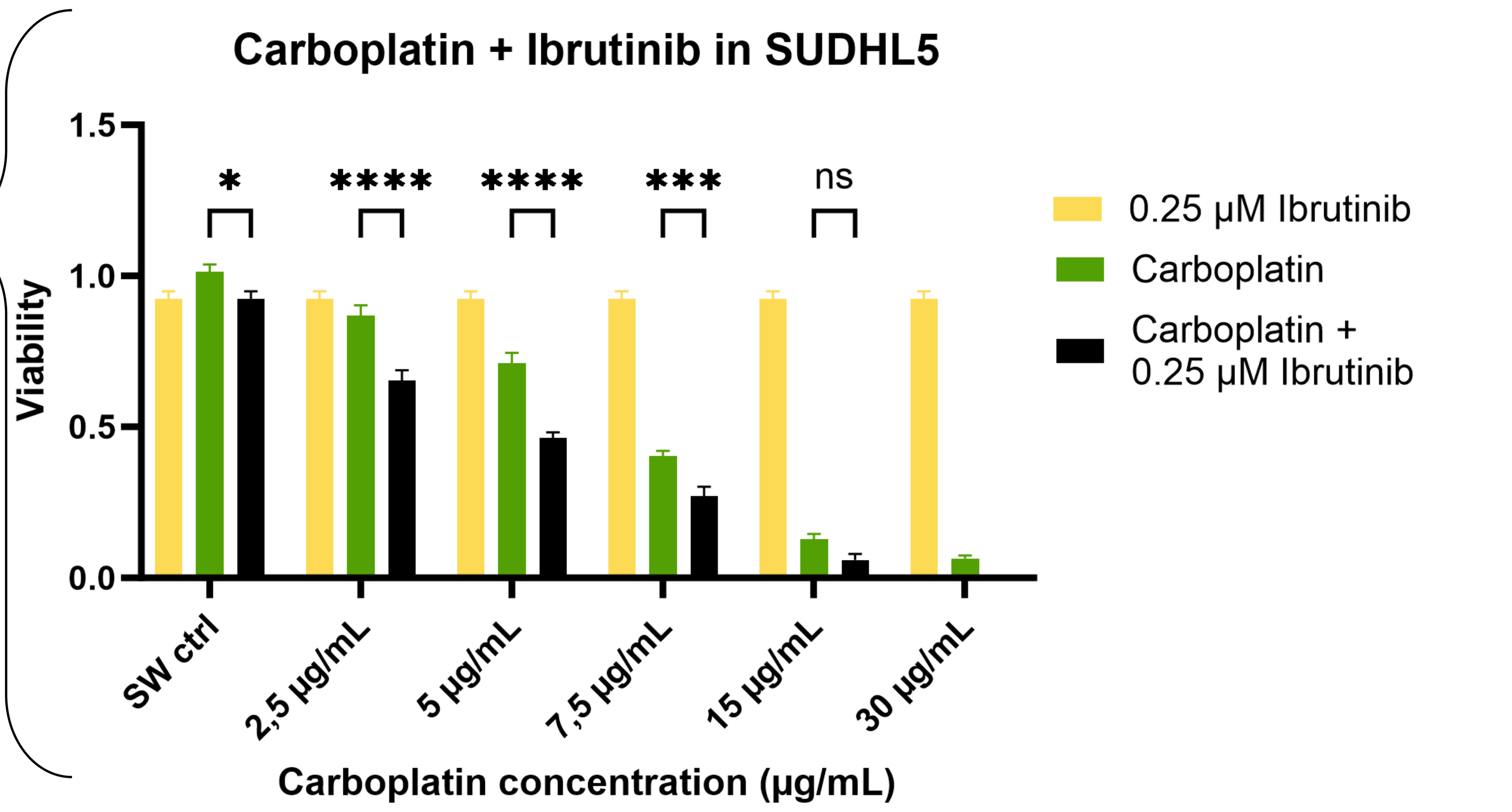

Figure S6

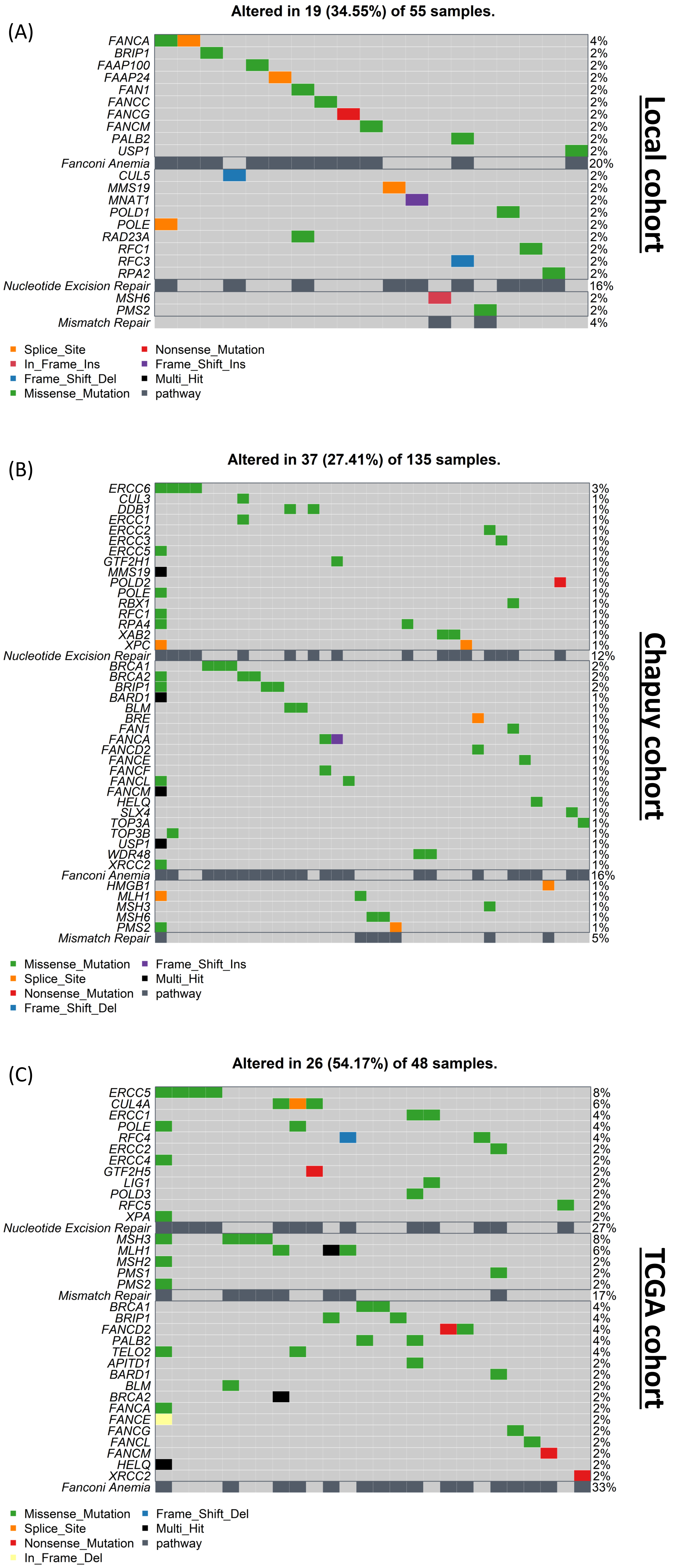
(A)
Altered in 19 (34.55%) of 55 samples.
FANCA 4%
BRIP1 2%
FAAP100 2%
FAAP24 2%
FAN1 2%
FANCC 2%
FANCG 2%
FANCM 2%
PALB2 2%
USP1 2%
Fanconi Anemia 20%
CUL5 2%
MMS19 2%
MNAT1 2%
POLD1 2%
POLE 2%
RAD23A 2%
RFC1 2%
RFC3 2%
RPA2 2%
Nucleotide Excision Repair 16%
MSH6 2%
PMS2 2%
Mismatch Repair 4%
Splice_Site
In_Frame_Ins
Frame_Shift_Del
Missense_Mutation
Nonsense_Mutation
Frame_Shift_Ins
Multi_Hit
pathway
Local cohort
(B)
Altered in 37 (27.41%) of 135 samples.
ERCC6 3%
CUL3 1%
DDB1 1%
ERCC1 1%
ERCC2 1%
ERCC3 1%
ERCC5 1%
GTF2H1 1%
MMS19 1%
POLD2 1%
POLE 1%
RBX1 1%
RFC1 1%
RPA4 1%
XAB2 1%
XPC 1%
Nucleotide Excision Repair 12%
BRCA1 2%
BRCA2 2%
BRIP1 2%
BARD1 1%
BLM 1%
BRE 1%
FAN1 1%
FANCA 1%
FANCD2 1%
FANCE 1%
FANCF 1%
FANCL 1%
FANCM 1%
HELQ 1%
SLX4 1%
TOP3A 1%
TOP3B 1%
USP1 1%
WDR48 1%
XRCC2 1%
Fanconi Anemia 16%
HMGB1 1%
MLH1 1%
MSH3 1%
MSH6 1%
PMS2 1%
Mismatch Repair 5%
Missense_Mutation
Splice_Site
Nonsense_Mutation
Frame_Shift_Del
Frame_Shift_Ins
Multi_Hit
pathway
Chapuy cohort
(C)
Altered in 26 (54.17%) of 48 samples.
ERCC5 8%
CUL4A 6%
ERCC1 4%
POLE 4%
RFC4 4%
ERCC2 2%
ERCC4 2%
GTF2H5 2%
LIG1 2%
POLD3 2%
RFC5 2%
XPA 2%
Nucleotide Excision Repair 27%
MSH3 8%
MLH1 6%
MSH2 2%
PMS1 2%
PMS2 2%
Mismatch Repair 17%
BRCA1 4%
BRIP1 4%
FANCD2 4%
PALB2 4%
TELO2 4%
APITD1 2%
BARD1 2%
BLM 2%
BRCA2 2%
FANCA 2%
FANCE 2%
FANCG 2%
FANCL 2%
FANCM 2%
HELQ 2%
XRCC2 2%
Fanconi Anemia 33%
Missense_Mutation
Splice_Site
Nonsense_Mutation
In_Frame_Del
Frame_Shift_Del
Multi_Hit
pathway
TCGA cohort
