# Supplementary material for: CRISPR-Cas9 Knockout Screens Identify DNA Damage Response Pathways and BTK as Essential for Cisplatin Response in Diffuse Large B-Cell Lymphoma
Source: Cancers (Basel). 2024 Jul 2;16(13):2437. doi: 10.3390/cancers16132437 (PMC11240649; doi:10.3390/cancers16132437)

Figure 2A – original Western blot gels

For HBL1 (H), OCILY7 (O), and RIVA (R)

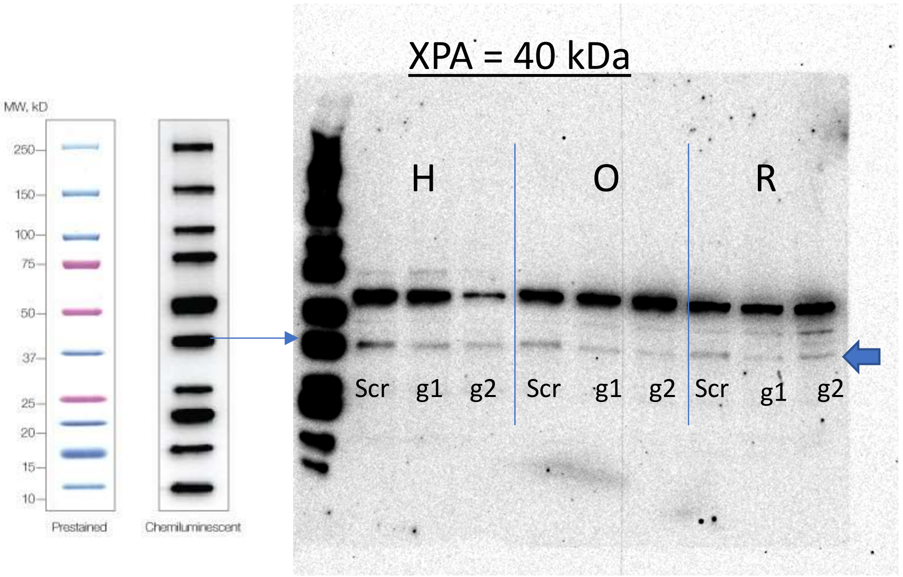

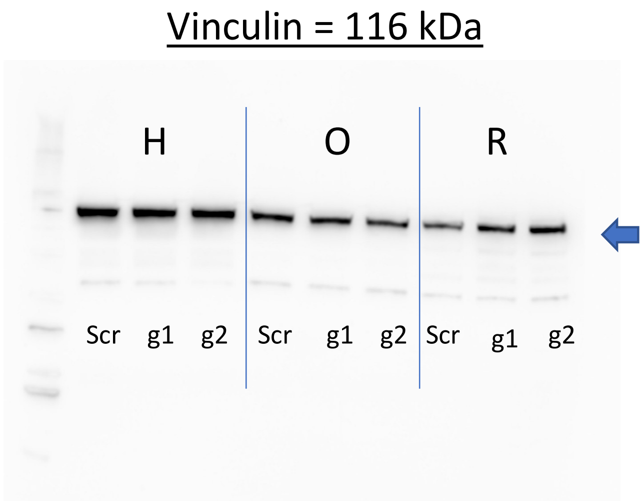

Figure 2A cont'd – original Western blot gels

For SUDHL5 (S) [and RIVA (R) again, but unused)

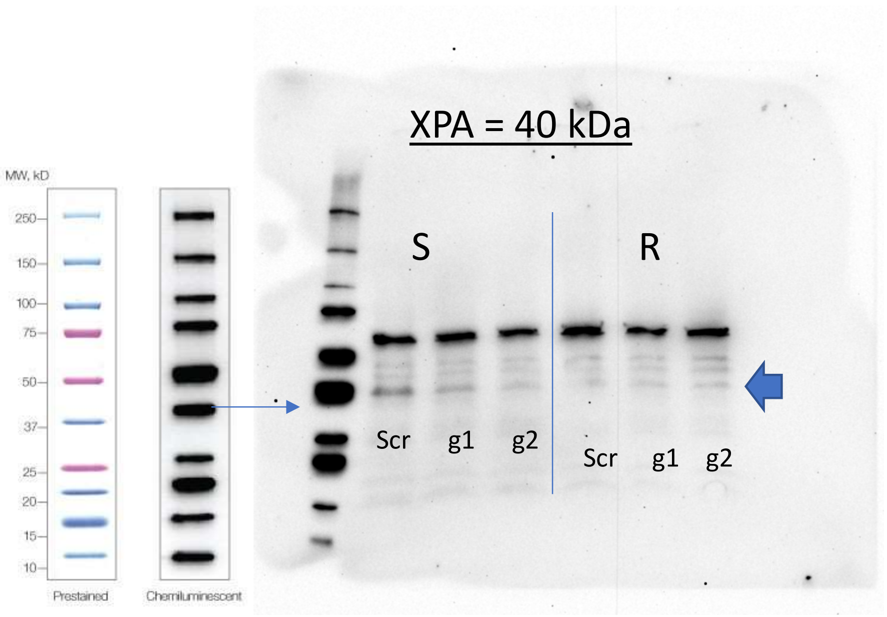

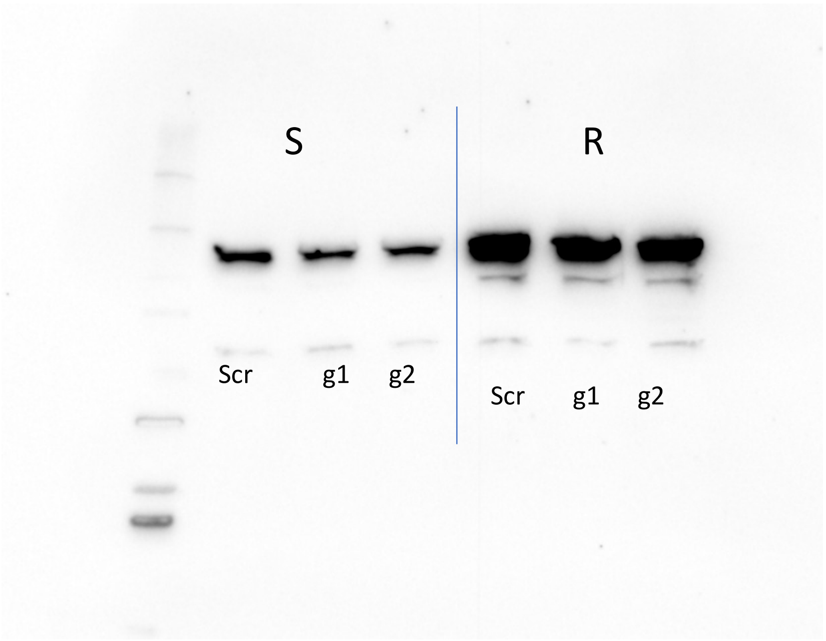

Figure 3A – original Western blot gels

ERCC6

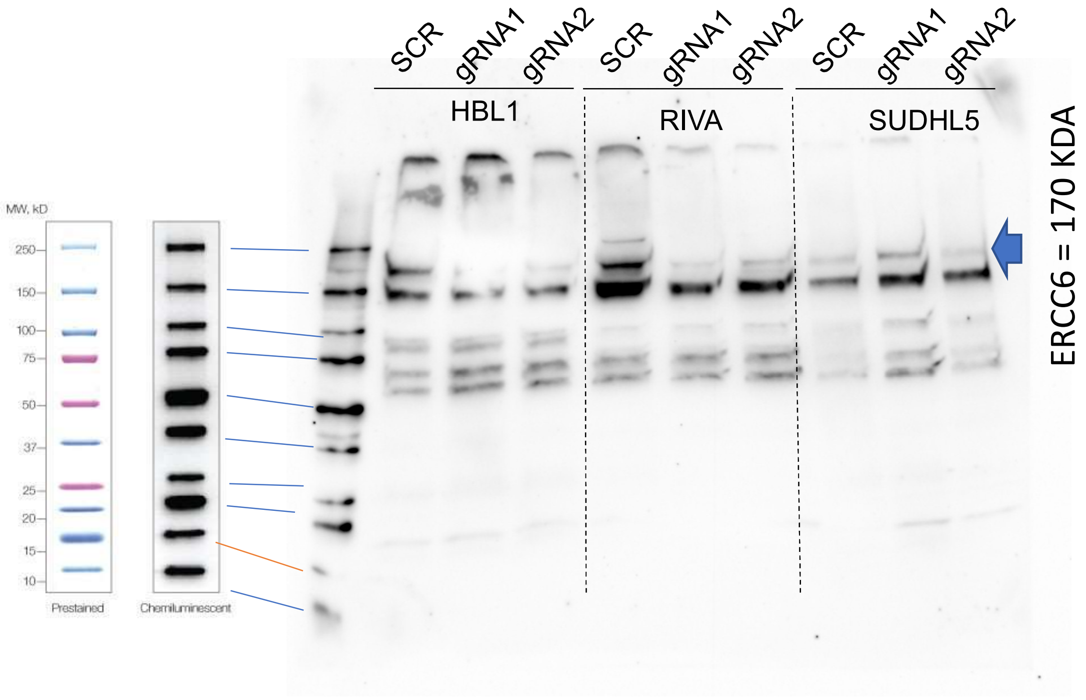

Beta actin

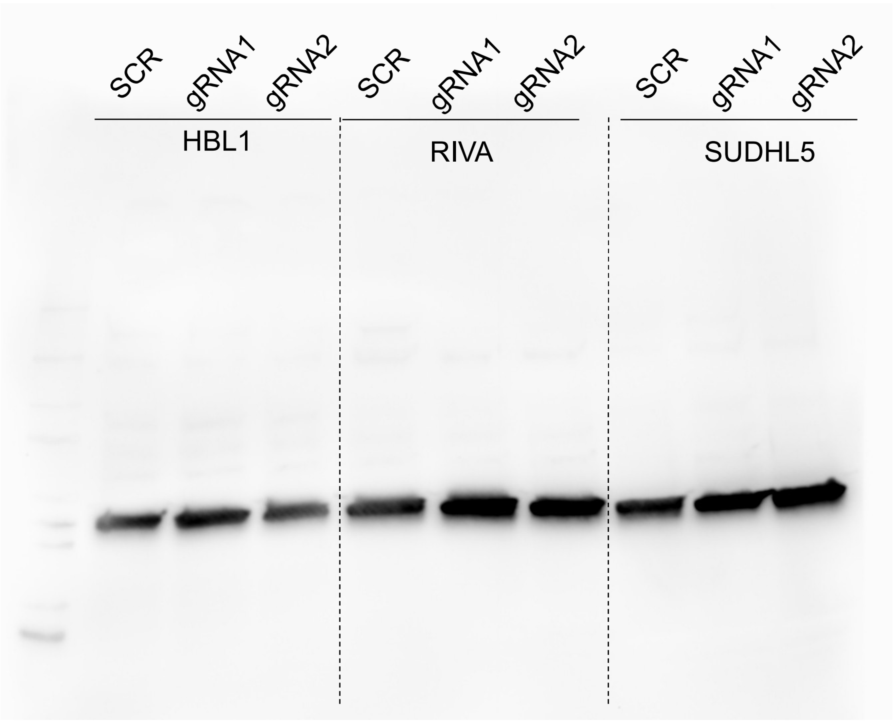

Figure 4A – original Western blot gels

BTK

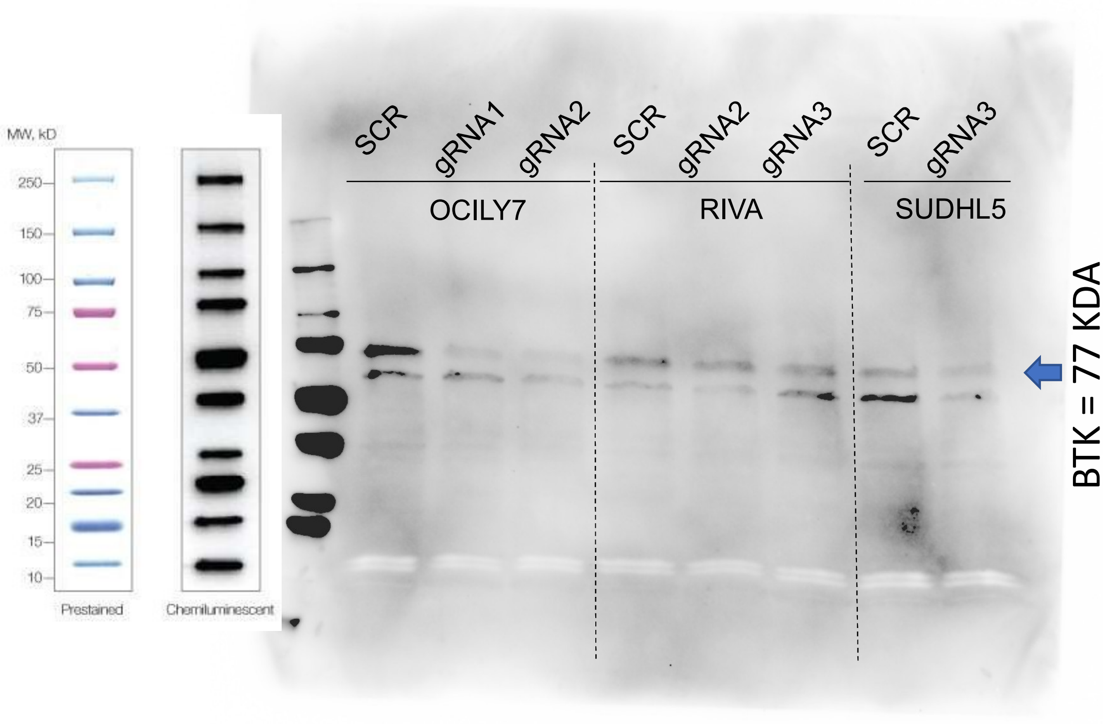

Beta actin

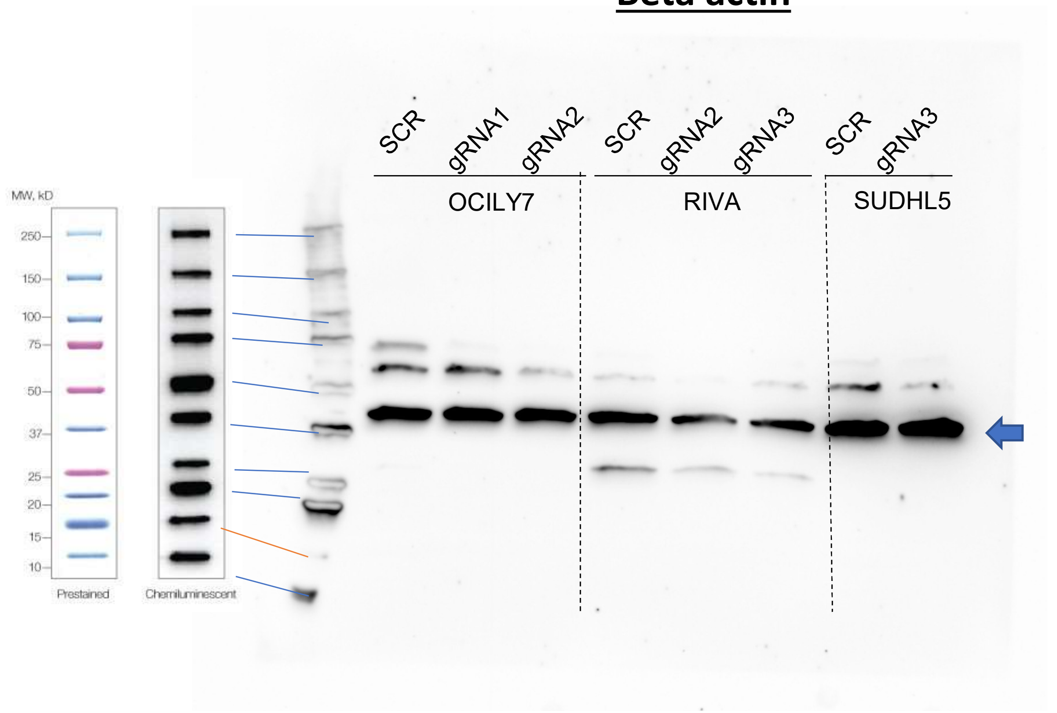

Supplement: Supplementary file 1 [file cancers-16-02437-s001.zip › Supplementary File S1. Original Western blot gels.pdf]
