# Supplementary material for: CRISPR-Cas9 Knockout Screens Identify DNA Damage Response Pathways and BTK as Essential for Cisplatin Response in Diffuse Large B-Cell Lymphoma
Source: Cancers (Basel). 2024 Jul 2;16(13):2437. doi: 10.3390/cancers16132437 (PMC11240649; doi:10.3390/cancers16132437)
Supplement: Supplementary file 1 [file cancers-16-02437-s001.zip › Supplementary materials and methods.pdf]

Supplementary materials and methods

DNA damage and cell cycle

To detect the level of DNA damage in cells with and without cisplatin treatment γH2AX were used as a marker. Cells were seeded at a concentration of $0.5x10^6$ cells/mL in four replicates in 12-well plates and treated with 0.85µg/mL cisplatin or vehicle (saline) for 24h prior. To ensure DNA damage was not caused by apoptosis, cells were harvested after 24h and counted on a NucleoCounter NC-200 (ChemoMetec, Allerod, Denmark) in which cells are labelled with acridine orange and DAPI to count total and dead cell populations, respectively. Highly viable cells (>97%) were fixed in 70% ethanol and samples were stored at -20˚C until analysis.

Prior to flow cytometry, samples were stained in a 1:40 ratio of FITC anti-H2A.X phosphor (ser139) (phospho-γH2AX) antibody (Biolegend, Cat#613,404) and staining buffer (BD Pharmingen™. Cat.No. 554657) (1h, 4˚C). Samples were analyzed on a cytometer (SONY, SH800 Cell Sorter). Unstained samples were used to gate for positive γH2AX signal.

For cell cycle experiments, untreated samples from γH2AX experiments were subsequently washed and treated with RNase A (Qiagen, Cat.No.19101) (30min, 37˚C) and stained with propidium iodide (BioLegend, Cat.No.421301) before flow cytometry (SONY, SH800 Cell Sorter).

For all flow cytometry experiments, gating strategy included removal of cell debris and doublets. Gates for positive γH2AX and PI signal was set using unstained cells. Flow cytometry experiments were run 2-3 times.

Western blot

Materials used for western blot are specified in table S3. Whole cell protein lysates were obtained using RIPA buffer and sonication (Diagenode, Biorupter Next Gen) at high pulse (6 x [30s on, 30s off]). Samples were subsequently incubated on ice (15min) and spun (15min, 14000xg). The supernatant was harvested, and protein concentration determined by a BCA protein assay according to the manufacturer's instructions. Three parts sample corresponding to 40µg of protein were mixed with one part 5% DTT-Laemmli. Samples were heated (5min, 100˚C) prior to loading in wells of an SDS-PAGE gel. The samples were run 10min at 50V and 25min at 250V. Next, proteins were transferred to a membrane, which was blocked in 5% skim milk (1h, 1000rpm). The membranes were incubated with primary antibodies, preferentially with epitopes downstream of the expected Cas9 cutsite (overnight, 1000rpm) and washed 3x before incubation with secondary antibody (anti-mouse or anti-rabbit) (2h, 1000rpm) conjugated with horse-radish peroxidase and

StrepTactin. The membranes were visualized through chemiluminescence using ECL reagent. The membranes were subsequently stripped and reprobed with primary antibody targeting the loading control (vinculin or β-actin) and proceeded as previously described.
